# Therapeutic Polymer-Based Cannabidiol Formulation: Tackling Neuroinflammation Associated with Ischemic Events in the Brain

**DOI:** 10.1021/acs.molpharmaceut.3c00244

**Published:** 2024-02-27

**Authors:** Merari Tumin Chevalier, Mansoor Al-Waeel, Amir M. Alsharabasy, Ana Lúcia Rebelo, Sergio Martin-Saldaña, Abhay Pandit

**Affiliations:** CÚRAM, SFI Research Centre for Medical Devices, University of Galway, Galway H92 W2TY, Ireland

**Keywords:** cannabidiol, stroke, neuroinflammation, oxygen and glucose deprivation, poly(lactic-*co*-glycolic acid)

## Abstract

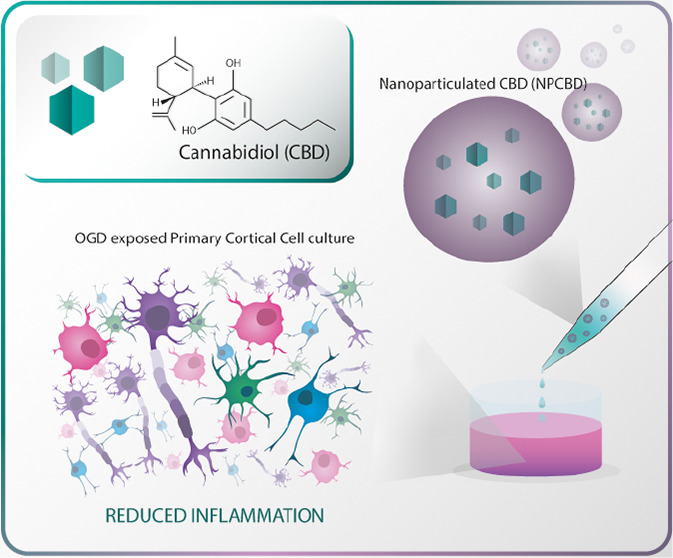

Cannabidiol (CBD) is the most relevant nonpsychostimulant phytocompound found in *Cannabis sativa*. CBD has been extensively studied and has been proposed as a therapeutic candidate for neuroinflammation-related conditions. However, being a highly lipophilic drug, it has several drawbacks for pharmaceutical use, including low solubility and high permeability. Synthetic polymers can be used as drug delivery systems to improve CBD’s stability, half-life, and biodistribution. Here, we propose using a synthetic polymer as a nanoparticulate vehicle for CBD (NPCBD) to overcome the pharmacological drawbacks of free drugs. We tested the NPCBD-engineered system in the context of ischemic events in a relevant oxygen and glucose deprivation (OGD) model in primary cortical cells (PCC). Moreover, we have characterized the inflammatory response of relevant cell types, such as THP-1 (human monocytes), HMC3 (human microglia), and PCC, to NPCBD and observed a shift in the inflammatory state of the treated cells after the ischemic event. In addition, NPCBD exhibited a promising ability to restore mitochondrial function after OGD insult in both HMC3 and PCC cells at low doses of 1 and 0.2 μM CBD. Taken together, these results suggest the potential for preclinical use.

## 1 Introduction

Stroke is the leading cause of long-term disability and the second leading cause of death worldwide. Of all the stroke cases, 7.6 million corresponds to ischemic stroke, leading to 3.3 million deaths annually.^1^ Ischemic stroke is caused by a blood supply interruption to a part of the brain.^1^ Patients may be eligible to receive intravenous thrombolysis treatment if they arrive at the hospital within 4.5 h from witnessed symptom onset; thrombolysis may be offered alone or in combination with endovascular thrombectomy, which has a 6 h time window for most patients.^2^ Thus, most stroke patients remain without any effective treatment option besides physical therapy. The human brain is incapable of efficient self-regeneration, which prevents full long-term functional recovery. Although insufficient, new therapies addressing brain damage due to ischemic stroke can boost the endogenous repair mechanisms of our brain. These approaches can lead to a pivotal step within a clinical scenario.

In the last two decades, the endocannabinoid system (ECS) has become a subject of great interest in neurobiology and neuropharmacology as a therapeutic target, mainly due to its wide distribution in the central nervous system.^3^ Indeed, it is particularly interesting when neuroinflammation plays a vital role in the onset of the disease component, such as stroke.^4^ Cannabidiol (CBD) is one of the hundreds of phytocannabinoids in the *Cannabis sativa* plant.^5^ CBD is a nonpsychostimulant but displays several beneficial pharmacological effects.^6^ Exhibiting a robust antioxidant and anti-inflammatory activity, glutamate release reduction, mitochondrial membrane stabilization, adenosine extracellular concentration increment, and nuclear factor kappa B (NF-κB) activation prevention, CBD has emerged as a solid neuroprotective candidate for the multifactorial treatment of ischemic stroke.^7^ However, to take advantage of the therapeutic properties of CBD, it is critical to address the intrinsic limitations preventing its effective delivery.^8^ CBD is highly lipophilic, which results in the use of high doses to achieve a therapeutic effect due to its poor biodistribution, also causing undesired side effects.^8^ The most preferred route for CBD administration is the oral route, but the oral bioavailability of CBD is estimated at 6% due to gastrointestinal precipitation exhibited by highly lipophilic drugs.^9^ Moreover, the available CBD preparations include oil- and alcohol-based solutions, which can easily lead to irritation and local inflammatory responses.^9^ CBD is highly sensitive to light and temperature and easily undergoes oxidation and degradation.^9,10^ Recent studies have reported mild to severe side effects related to the need of increasing CBD doses to achieve therapeutic efficacy both in animals^11^ and humans.^12^ Therefore, a simplistic and feasible clinical translation approach is required to extend CBD’s effective delivery and reduce dosages by increasing the drug’s bioavailability. The degradation of CBD in gastrointestinal conditions and the first-pass metabolism of the liver can be avoided if intravenous administration is considered.^13^

**Scheme 1:**
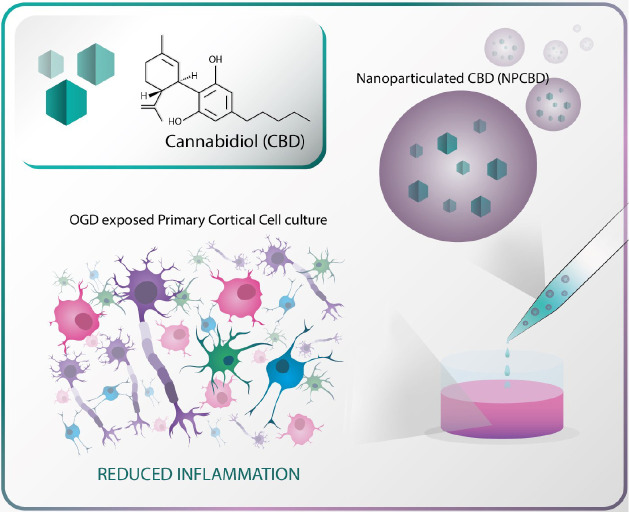
Cannabidiol (CBD)-Loaded Nanoformulation (NPCBD) Schematic and Overview of the *In Vitro* Protective Effect Reducing inflammation After Oxygen and Glucose Deprivation (OGD) Over Primary Cortical Cells (PCC) Culture

Polymeric systems have been used for drug delivery, and among them, biodegradable polymeric nanoparticles (NPs) can efficiently accommodate hydrophobic drugs and deliver them opportunely. Herein, we have used poly(lactic-*co*-glycolic acid) (PLGA)-based NPs to confer efficiency and versatility to CBD delivery. The selected polymer serves as the NP matrix and overcomes pharmacological drawbacks and is a well-established, FDA-approved polyester able to be nanostructured in a reproducible manner with a high CBD loading efficiency. Herein, we aim to demonstrate that our engineered CBD-loaded PLGA NPs (NPCBD) are safe to use and that CBD’s availability *in vitro* is as good as CBD in its classic formulations, with improved physiochemical properties from a pharmacological point of view. We have successfully fabricated CBD-loaded PLGA NPs (NPCBD) and tested them in relevant *in vitro* models of human monocytes (THP-1) and microglia (HMC3). Moreover, we have evaluated NPCBD therapeutic performance in ischemic events exposing a primary cortical cells (PCC) to oxygen and glucose deprivation (OGD) conditions (Scheme 1). NPCBD improved not only in terms of metabolic activity and dsDNA content but also in the inflammatory state of the treated cells after OGD to a pro-regenerative one, suggesting a potential for its preclinical use.

## 2 Methodology

### 2.1 Fabrication of Therapeutic Polymer-Based Cannabidiol Nanoformulations

CBD-loaded PLGA NPs (NPCBD) were prepared by single emulsion method with subsequent solvent evaporation as previously reported.^14^ The NPs were collected by centrifugation at 11,000*g* after four washes with deionized water to remove any excess surfactant.^14^ Briefly, 50 mg of PLGA and 5 mg of CBD were dissolved in 2.5 mL of dichloromethane (DCM) by using magnetic agitation. This solution was emulsified with 2% w/v of PVA aqueous solution using a tip sonicator (VIBRA CELL Sonics mod. VC 750 USA, 39% Amp) for 20 min. The resulting emulsion was transferred into a 0.2% (w/v) PVA aqueous solution, which was then magnetically stirred at room temperature to enhance complete solvent evaporation. Unloaded PLGA NPs (NP-0) were obtained by using the same technique.

### 2.2 Characterization of Cannabidiol-Loaded Nanoparticles

#### 2.2.1 Morphology

The morphology of NPCBD was analyzed by a field emission scanning electron microscope (FESEM, Hitachi SU 8000 TED). Samples were prepared by placing freeze-dried NPs over a carbon tape disk with subsequent coating with a gold–palladium alloy (80:20).

#### 2.2.2 Particle Size Distribution

Measurements of NPCBD dispersions were performed in square polystyrene cuvettes (SARSTEDT), and the temperature was kept constant at 25 °C. The particle size distribution of the nanoparticulate dispersions was determined by dynamic light scattering (DLS) using a Malvern Zetasizer. The zeta potential (ζ) was determined for NP formulations at 1 mg/mL PBS concentration and pH = 7.4, which is automatically calculated from the electrophoretic mobility using Smoluchowskí’s approximation. The evolution of the mean diameter with the temperature from 24 to 40 °C was also evaluated in the same apparatus. The statistical average and standard deviation of data were calculated from 6 different samples (3 measurements of 20 runs each one).

#### 2.2.3 Loading Efficiency

The loading efficiency (LE%) of CBD in the PLGA NPs was determined by spectroscopy using the NanoDrop 2000c spectrophotometer (λ = 207 nm). Briefly, the amount of entrapped CBD was calculated indirectly by measuring the difference between the initial amount of CBD (CBDin) and free CBD (CBDfree) in the supernatant during the washing steps. The LE% was expressed according to eq 1.


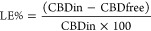
1

#### 2.2.4 Fourier-Transform Infrared Spectroscopy (FTIR)

FTIR was used to study the characteristic bands of all of the constituents of the proposed NPCBD. Infrared spectroscopy was performed in the attenuated total reflection mode on an IR 640 spectrophotometer (Varian). Samples were analyzed at room temperature by 16 scans using a resolution of 4 cm^–1^.

#### 2.2.5 CBD Stability Over Time

To determine the stability over time of CBD and NPCBD, the same amount of CBD in NPCBD or as a free drug was resuspended or dissolved in ACSF (with 20% DMSO in the case of the free drug) and incubated at 37 °C. At specific time points (3, 7, and 21 days), the supernatants were analyzed by HPLC to determine the amount of CBD remaining.

### 2.3 *In Vitro* Evaluation of Cannabidiol-Loaded Nanoparticles

#### 2.3.1 Monocytes (THP-1) and Microglia (HMC3)

THP-1 monocyte cell line, established from peripheral blood acute monocytic leukemia, was purchased from ATCC. To differentiate THP-1 into macrophages, 100 ng/mL phorbol 12-myristate 13-acetate (PMA) was used on a monocyte cell suspension. 4 ×10^5^ cells per well were seeded into 24-well plates with PMA-conditioned media for 24 h. After the differentiation, the media were refreshed with RPMI medium, and the cells were treated with lipopolysaccharide (LPS) (100 ng/mL) or different dilutions of NPCBD ranging from 100 to 5 μg/mL. Cells were exposed to the conditioned media for 72 h to determine the effect, if any. After the stimulation period, the culture medium was recovered and stored at −20 °C for further analysis.

The human microglia HMC3 cell line is a microglial cell isolated from a patient’s brain and purchased from ATCC. 4 ×10^5^ cells per well were seeded into 24-well plates. Cells were exposed to the conditioned media for 72 h to determine the effect, if any. After 24 h, cells were treated with LPS (100 ng/mL) or different dilutions of NPCBD ranging from 100 to 5 μg/mL. After the stimulation period, the culture medium was recovered and stored at −20 °C for further analysis.

#### 2.3.2 Primary Cortical Cell (PCC) Culture

Sprague–Dawley rat pups (P3) were sacrificed with modifications of the protocol published by Mathew et al.^15^ Cortex was harvested from the brain and chopped using a Mc tissue chopper. The extracted tissue was then incubated in a papain-based dissociation solution for 1 h to proceed with further trituration, strain, centrifugation, and cell counting. Research and animal procedures were performed in accordance with the European (EU) guidelines (2010/63/UE) and Health Products Regulatory Authority of Ireland. Every effort was made to minimize animal suffering and to reduce the number of animals used.

#### 2.3.3 Metabolic Activity and dsDNA Content Evaluation

The cell metabolic activity was evaluated by using the alamarBlue protocol (Thermofisher). Briefly, 300 μL of 10% alamarBlue was added per well and incubated for 3 h at 37 °C (5% CO_2_, humidity), and the solution was measured using a plate reader (Thermofisher, λex 545 nm, λem 590 nm).

Cell dsDNA content was evaluated by using the PicoGreen assay. Briefly, 100 μL of ultrapure water was added to each well, and after three cycles of freeze–thaw, the samples were used following the manufacturer’s protocol (PicoGreen Assay, Protocol ND-3300, Thermo Scientific). The ratio of metabolic activity per dsDNA content was calculated. Both determinations were performed eight times on the same cell culture experiment and were done in three independent experiments (*N* = 3).

#### 2.3.4 Inflammatory Response Induced by NPCBD by Human Multiplex ELISA

THP-1 or HMC3 response after NPCBD exposure was measured by ELISA according to the manufacturer’s specification (Meso Scale Diagnostics). Briefly, pro-inflammatory (TNF-α, IL-1β, IFN-γ, IL-6, IL-8 and IL-12) and anti-inflammatory (IL-10, IL-13 and IL-4) cytokines were quantified in the recovered culture media after NPCBD treatment for 3 days. Two technical replicates from 3 independent experiments were assessed.

### 2.4 Oxygen and Glucose Deprivation (OGD) Model Over PCC and HMC3

#### 2.4.1 HMC3 and PCC OGD Induction

This procedure was performed after culture of PCCs for 10 days or HMC3 for 1 day. Each well was rinsed twice with prewarmed OGD medium prepared according to Tasca et al.^16^ The medium in each well was replaced with an OGD medium, and the plate was left in the hypoxia chamber (N_2_ 95%/CO_2_ 5%) for 6 h. After OGD exposure, culture plates were removed from the hypoxia chamber; the wells were withdrawn from the OGD medium, replenished with a complete culture medium, and incubated for another 24 h. The control groups were cultured in parallel to those in the OGD group. Cellular damage was evaluated 24 h posterior to OGD exposure to emulate reperfusion.

#### 2.4.2 Rat Multiplex ELISA and Proteome Profiling

PCC and OGD-exposed PCC responses after NPCBD treatment were studied by multiplex ELISA according to the manufacturer’s specification (MSD). Briefly, pro-inflammatory (TNF-α, IL-1β, IFN-γ, IL-6, IL-8, and IL-12) and anti-inflammatory (IL-10, IL-13, and IL-4) cytokines were quantified in the recovered culture media after 1 day. Two technical replicates from three independent experiments were assessed.

The relative expression of chemokines, pro- and anti-inflammatory cytokines, and growth factors secreted by PCCs and OGD-exposed PCCs after NPCBD treatment was determined by the Rat XL Cytokine Proteome Profiler Array kit as per manufacturer’s instructions (ARY030, R and D SYSTEMS). The signals relative to protein expression were recorded on X-ray films (CL-XPosure Film, Thermo Scientific, 34090), which were further analyzed using Image Studio Lite software as pixel density. Furthermore, each analyte calculated the ‘mean pixel density,’ and the data were plotted as a heatmap. Hierarchical clustering of the data was performed using Morpheus (https://software.broadinstitute.org/morpheus).

#### 2.4.3 Immunocytochemistry

PCC cultures intended to be analyzed by image were seeded into poly-l-lysine (PLL)-coated coverslips in 24-well plates, and the coverslips received the same treatments described before and fixed at the desired time points (3 and 7 days post-treatment). Briefly, media was removed, and cells were washed with PBS, exposed for the desired time to 10% formalin, and washed again after fixation. Fixed cells were permeabilized by incubating them in 0.2% Triton X-100 in PBS, followed by washing with PBS. Nonspecific binding was blocked with 1% bovine serum albumin (BSA) in PBS solution for 1 h at room temperature (RT). Primary antibodies mouse anti-GFAP (1:500, Sigma-Aldrich), rabbit anti-Iba-1 (1:200, Dako), mouse anti-Olig-2 (1:200, Sigma-Aldrich), and rabbit anti-β-Tubulin III (1:500, Abcam) were prepared in blocking buffer and added to the plates, which were incubated overnight at 4 °C under stirring. After washing, cells were incubated with secondary antibodies Alexa Fluor 488 (1:500, Thermo Scientific, A-10667) and Alexa Fluor 546 (1:500, Thermo Scientific, A11035) for 1 h at RT. After washing, cells were incubated in DAPI (1:2000, Thermo Scientific) for 5 min at RT, and coverslips were mounted onto glass slides using a fluoromount and observed under a confocal microscope (Olympus FluoView 3000 system).

#### 2.4.4 Mitochondrial Function

The mitochondrial functions of PCC or HMC3 were assessed using the Cell Mito Stress Test by the measurement of oxygen consumption rate (OCR) and extracellular acidification rate (ECAR) of cells in real time using an XFp extracellular flux analyzer (Seahorse Bioscience, Agilent technologies, U.K). 2.5 × 10^4^ of PCC or HMC3 wells was seeded into each well of the 8-well Seahorse XFp cell culture mini plates (Agilent technologies). After growing in an incubator at 37 °C in 5% CO_2_ and reaching a confluence of 90%, cells were exposed to the OGD, as described in Section 2.4.1 and then treated with NPCBD for 3 days until assessment. On the selected end points, the media were replaced with unbuffered phenol red-free Seahorse XF base medium (DMEM) and incubated for 1 h at 37 °C without % CO_2_. The working medium was prepared freshly by the addition of glucose (25 mM), sodium pyruvate (1.0 mM), and l-glutamine (2.0 mM) to the medium and adjusting the pH at 7.4. The bioenergetic profiles were generated by sequential injection of multiple mitochondrial toxins resuspended in Seahorse assay medium: 1) oligomycin A (an ATP synthase inhibitor that blocks its proton channel/complex V), 2) carbonyl cyanide-4-(trifluoromethoxy)phenylhydrazone (FCCP; an uncoupling agent that disrupts the mitochondrial membrane potential by transporting hydrogen protons through the membrane and leading the respiration rate to its maximum capacity), and 3) rotenone/antimycin A (complex I and III inhibitors, respectively, which shut down mitochondrial respiration). The final concentrations of oligomycin A and rotenone/antimycin A in each well, after injection, were 1 μM and 0.5 μM, respectively in both types of cells. However, following FCCP titration, the optimized final concentration of FCCP was 0.5 and 1.5 μM in case of HMC3 and PCC cells, respectively. At the end of the assay, a graph and data sheet were generated in the system, allowing for the analysis of the effect of the different compounds on mitochondrial function. Four independent experiments calculated parameters such as basal, ATP-linked, and reserve capacity OCRs from the Mito Stress assays.

### 2.5 Statistical Analysis

Graphs and figures were created by using GraphPad Software. Statistical differences in histochemical quantification were analyzed by GraphPad Software. One-way ANOVA was performed, followed by Tukey’s or Dunnet’s posthoc test where appropriate. Mean and SEM were calculated for all groups. All error bars indicate SEM.

## 3 Results and Discussion

### 3.1 CBD Was Efficiently Accommodated into the PLGA Matrix, and the Resulting NPCBD Exhibited Suitable Properties for CBD Delivery

**Figure 1:**
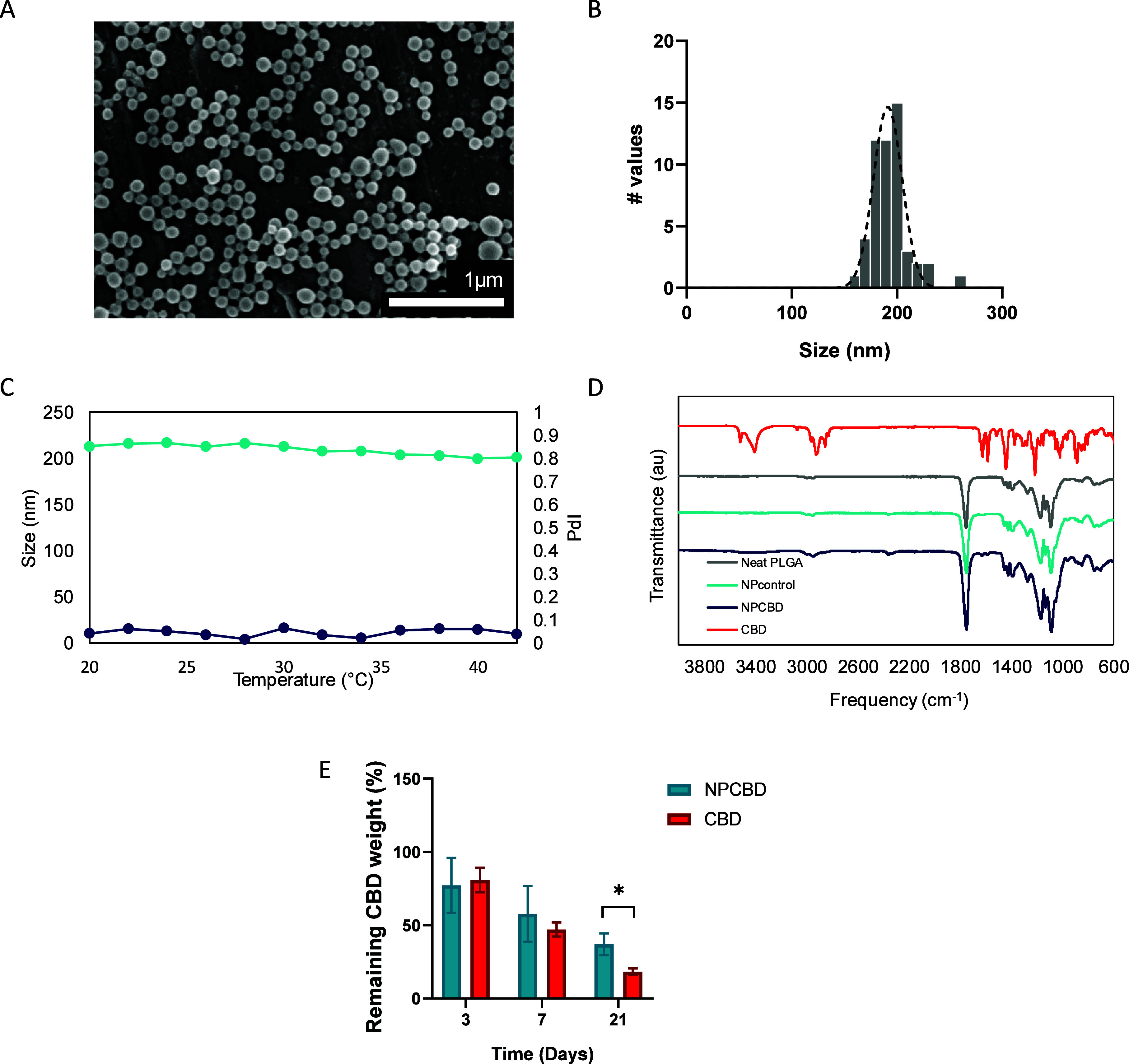
NPCBD possesses suitable physicochemical properties for enhancing CBD pharmacological properties. (A) FESEM micrographs of NPCBD; (B) extracted histogram of NPCBD distribution; (C) NPCBD stability showed in terms of the hydrodynamic diameter in nm, and the polydispersity index (PDI) in a range of temperatures from 20 to 42 °C; (D) FTIR spectra of the neat PLGA, NP0, NPCBD, and the free drug; (E) stability of free CBD when compared to NPCBD up to 21 days incubated in ACSF at 37 °C. Data expressed as % of CBD remaining analyzed by HPLC (data are represented as mean ± SEM, *N* ≥ 4 experimental replicates. *p < 0.05, one-way ANOVA followed by Tukey’s posthoc test).

Among all the new therapeutics proposed for managing ischemic stroke, CBD arose as a promising pharmacological ingredient due to its anti-inflammatory, immunosuppressive, and analgesic properties.^7^ However, CBD’s high hydrophobicity limits its clinical implementation due to the use of high doses needed to achieve a therapeutic effect.^17^ To overcome CBD pharmacological drawbacks, polymer carriers can be an innovative strategy to improve efficacy and reduce side effects while maintaining the properties of the entrapped active substances.^18^ We deliberately selected a well-studied and widely used polymer to build our CBD carriers due to the enhanced feasibility for clinical translation. PLGA has a well-known ability to efficiently accommodate hydrophobic drugs through the solvent evaporation method, and it has an appealing history with FDA regulators. PLGA was nanostructured through a single emulsion/solvent evaporation technique, yielding NPCBD. FESEM images of the obtained NPs exhibited a quasi-spherical morphology (Figure 1A,B) associated with effective polymer processing without noticeable loss of the droplet structure during the first stage of freeze-drying.^14^ The fundamental features of the proposed CBD carriers are summarized in Table 1. DLS measurements of NPCBD evidenced a hydrodynamic diameter of 240 nm and a zeta potential value of −21.5 ± 0.60 mV due to the intrinsic negative charges in the PLGA chemical structure. Moreover, the particles exhibited a narrow distribution, which is confirmed by DLS with a PDI of 0.15 ± 0.030. Differences among the sizes exhibited in FESEM micrographs and DLS measurements are typical in this type of system and result from the hydration and swelling of the NPs in aqueous dispersion (Figure 1B). Furthermore, the ability of PLGA to efficiently accommodate and trap hydrophobic molecules can be observed again with the NPCBD, showing a LE of 82% of the initial CBD amount. This high loading efficiency is directly related to the hydrophobic nature of both the polymer and drug. Values of hydrodynamic diameter, zeta potential, and PDI indicate monodispersity, nonaggregation, and colloidal stability (Table S1). In addition, these values show the feasibility of using NPCBD for intravenous administration, which is confirmed by the redispersion assay showing that both the Dh and PDI remain practically unchanged considering possible temperature ranges according to the physiological medium.

**Table 1:** NPCBD Physicochemical Properties, Including Hydrodynamic Diameter (Dh, by Intensity), Polydispersity Index (PDI); Zeta Potential (δ), Loading Efficiency (LE%), and Mass of CBD Per mg of NPs Expressed in μg/mg NPs

ID	CBD % w/w	Dh (nm)	PDI	δ (mV)	LE%	CBD μg/mg NPs
CBD-loaded NPs	10	240.1 ± 10.1	0.15 ± 0.03	–21.5 ± 0.60	82	63
Unloaded NPs	-	261.6 ± 10.2	0.16 ± 0.02	–24.1 ± 0.76	-	-

To provide information regarding the stability of NPCBD in the presence of potential temperature changes, the hydrodynamic diameter and PDI evolution were evaluated by DLS over a temperature range according to the human biological milieu (Figure 1C). Both parameters remained constant, indicating that NPCBD possesses adequate thermal behavior for the proposed application showing good stability with minor changes at temperatures ranging from 20 to 42 °C.

ATR-FTIR was used to determine the chemical identity and interaction between CBD and PLGA in the resulting NPCBD (Figure 1D). It was noted that CBD and NPCBD shared a common spectral doublet feature between 1580 and 1620 cm^–1^, with the peak center and the width of the band being slightly different among them. The double peak is centered at 1583 and 1627 cm^–1^ for free CBD and 1585 and 1622 cm^–1^ for NPCBD. These bands arise from the C=C stretching vibration of the aromatic ring and the C=C stretches in the cyclohexane ring of CBD, confirming the presence of the drug in the system.

Moreover, a study of the system’s stability in suspension in ACSF was performed to study how the polymeric particle protects the integrity of CBD. No differences among free CBD and NPCBD were observed during the first week (Figure 1E). However, it can be noticed that the amount of CBD remaining in the formulation after 21 days of incubation was considerably higher in the NPCBD, with 37% CBD remaining, compared to 18% on the free CBD. Thus, our engineered system shows enhanced stability provided by the polymeric particle entrapping the CBD, potentially increasing its bioavailability in biological fluids.

### 3.2 NPCBD Induced an Amenable Inflammatory Profile Over THP-1 and HMC3

**Figure 2:**
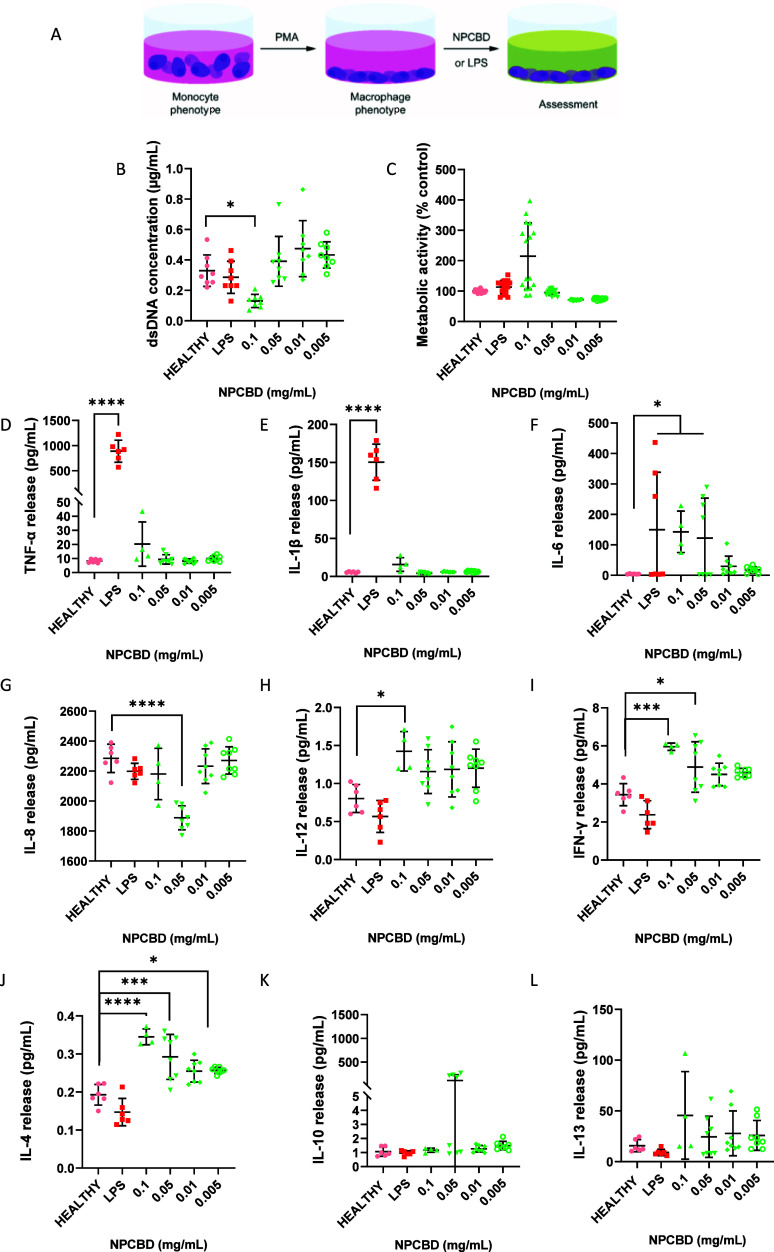
NPCBD treatment for 3 days did not induce significant changes in the THP-1 inflammatory state. (A) Experimental workflow of in THP-1 *in vitro* model. (B) dsDNA concentration was determined by Picogreen analysis, and (C) metabolic activity of the culture was determined by alamarBlue after 3 days of treatment with NPCBD. Determination of the release of pro-inflammatory (D–I) and anti-inflammatory (J–K) cytokines from the THP-1 cells after 3 days of treatment with NPCBD at four doses (mg/mL). Cytokine concentration released to the supernatant was analyzed by multiplex ELISA. Data are represented as mean ± SEM, *N* ≥ 4 experimental replicates. *p < 0.05, **p < 0.01, ***p < 0.001 vs Healthy cells. One-way ANOVA followed by Tukey’s posthoc test.

It is crucial to know the potential toxic effect of our NPCBD on relevant cell populations such as macrophages (THP-1) and microglia (HMC3) to ensure a suitable immune response. To study any potential detrimental immune response, we assessed NPCBD toxicity on THP-1 cells, a monocyte-like cell line derived from the blood of a young patient with acute monocytic leukemia, which is a robust tool for studying monocyte function and response in health and disease scenarios.^19^ The biological response was compared to that of the well-known pro-inflammatory molecule LPS, which triggers a robust and reproducible inflammatory response (Figure 2). THP-1 cells were exposed to 100 ng/mL LPS or NPCBD suspension at concentrations ranging from 0.1 to 0.005 mg/mL. In order to evaluate how cells respond to NPCBD, cultures were treated with the CBD formulation over 3 days (Figure 2A). No statistically significant differences were appreciated among groups in terms of metabolic activity and dsDNA content, but in the case of the highest concentration of NPCBD, a significant decrease from an average value of 0.33 to 0.13 μg/mL in dsDNA content was shown. Hence, data showed that 0.1 mg/mL NPCBD (corresponding to 2 μM CBD) exhibited a cytotoxic effect potentially due to the high concentration of the formulation for an *in vitro* application (Figure 2B). However, the cytotoxic effect can be mild even when NPCBD is used at a 1000× higher concentration than the LPS..

To study the potential immune and inflammatory response to NPCBD, we quantified the secretion of nine cytokines that play a pivotal role in ameliorating or worsening inflammation.^20^ The secreted biomarkers from monocyte-derived macrophages, such as the pro-inflammatory cytokines, TNF-α, IL-1β, IL-6, IL-8, IL-12, and IFN-γ (Figure 2D–I) and anti-inflammatory cytokines, IL-4, IL-10, and IL-13 (Figure 2H–L) were studied. The response of THP-1 to inflammatory insults such as LPS is widely reported, and as was expected, the cell line exhibited a pro-inflammatory stage similar to the one found in human monocyte-derived macrophages.^19^ When cells were treated with NPCBD, all of the groups presented a different cytokine phenotype than the LPS group. Notably, the LPS-treated group showed a significantly higher release of TNF-α of 11,900% (7 pg/mL compared to 833 pg/mL) and IL-1β of 2,980% (5 to 149 pg/mL) than NPCBD or Healthy (untreated) cells, suggesting a more amenable environment for the cells. However, for IL-6, NPCBD-treated groups secreted a significantly larger amount of 2,980% (5–149 pg/mL) of the cytokine than the healthy group, showing that the NPCBD might induce some inflammatory response through IL-6 upregulation. Recently, CBD’s ability to modulate IL-6 secretion in human monocytes after LPS-induced inflammation has been reported.^21^ In contrast with our findings, a downregulation was reported independent of the CBD dose (0.5, 1, and 10 μM CBD). However, the effect on cytokine secretion by CBD alone was not reported. In contrast, our results showed an apparent dose-dependent influence on IL-6 release, similar to that of LPS-treated cells when cells were exposed to 0.1 mg/mL (2 μM CBD) and 0.05 mg/mL (1 μM CBD). A sex-dependent increase in IL-6 release in healthy animals after CBD treatment has been reported to be considerably higher in healthy males.^22^ However, further studies are needed to characterize the effects of CBD on inflammatory cytokines in healthy donors and sex-related differences on IL-6 expression. A similar effect has been reported in IL-12 and IFN-γ related to Th1 activation and proliferation.^20^ However, these values are observed only in the two higher concentrations, while no differences were seen when cells were treated with lower doses of NPCBD (Figure 2H–I).

Regarding the anti-inflammatory cytokines, no significant differences were seen in any cytokine except IL-4 (Figure 2J–L). A significantly higher release of the anti-inflammatory IL-4, an increase of 200% (0.2 pg/mL compared to more than 0.4 pg/mL), was shown in all NPCBD groups. However, as seen with IL-12 and IFN-γ, the physiological relevance of these differences may be insignificant due to the detected low concentrations of the cytokine. Altogether, these results showed the potential safety of the engineered NPCBD, preventing an unwanted detrimental pro-inflammatory response. THP-1 cells are widely used as an *in vitro* model to study inflammatory signaling since their behavior resembles one of freshly isolated primary human peripheral blood monocyte-derived macrophages. However, it might be noticed that some changes in gene expression after LPS stimulation are different between them.^23^

**Figure 3:**
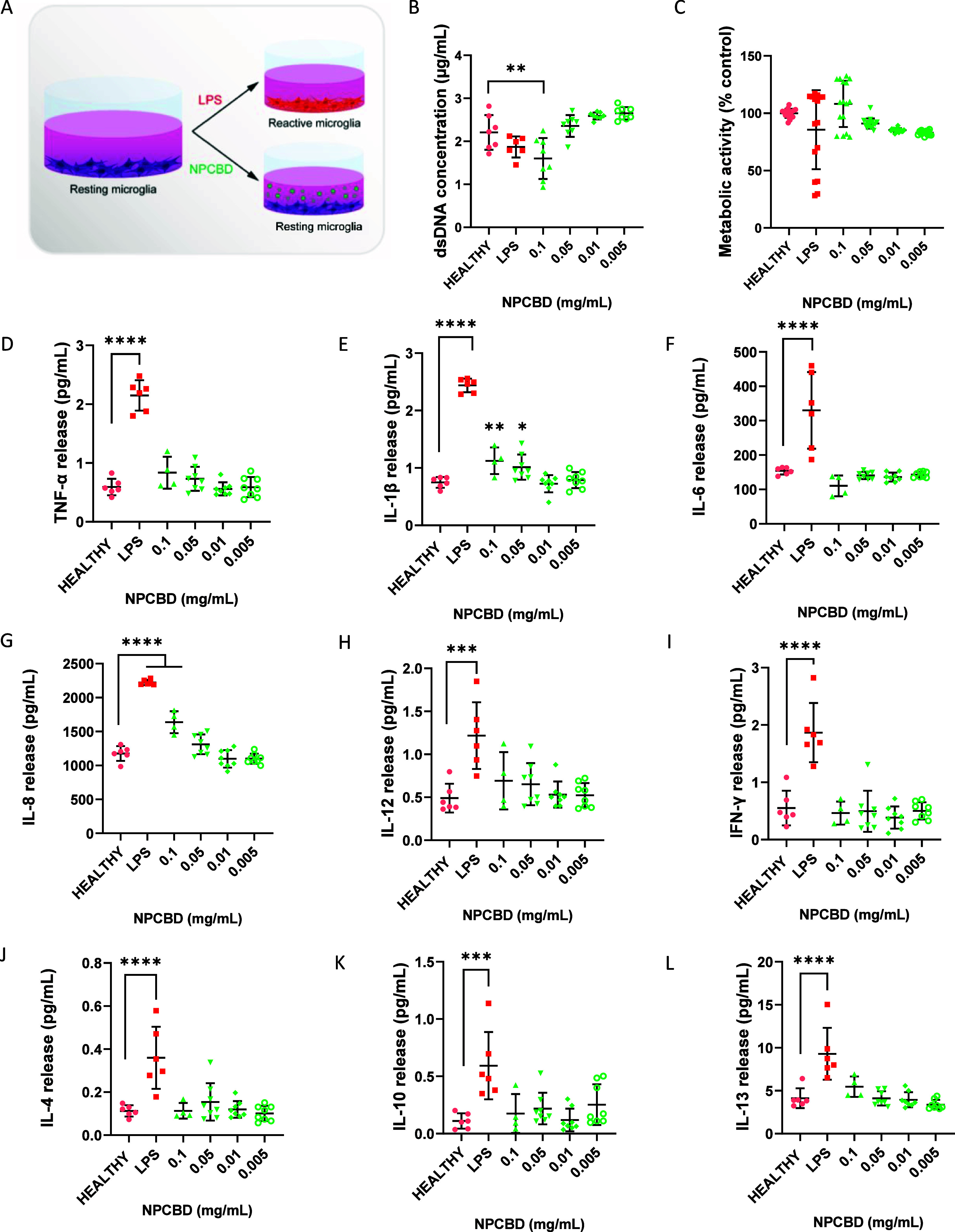
HMC3 inflammatory phenotype after NPCBD treatment for 3 days does not show significant differences with healthy cells. (A) Experimental workflow of HMC3 *in vitro* model. (B) dsDNA concentration was determined by Picogreen analysis and (C) metabolic activity of the culture was determined by alamarBlue after 3 days of treatment with NPCBD. Determination of the release of pro-inflammatory (D–I) and anti-inflammatory (J–K) cytokines from the HMC3 cells after 3 days of treatment with NPCBD at four doses. Cytokine concentration released to the supernatant was analyzed by multiplex ELISA. Data are represented as mean ± SEM, *N* ≥ 4 experimental replicates. *p < 0.05, **p < 0.01, ***p < 0.001 vs Healthy cells. One-way ANOVA followed by Tukey’s posthoc test.

Microglia are the first line of defense of the CNS, and their function and activation have been extensively studied in experimental models mainly in rodent ones.^24^ However, the characterization of human cells was limited due to the restricted availability of primary sources of human microglia. Hence, human immortalized microglial cell lines were developed being HMC3, previously named CHME3, the most reliable model nowadays.^25^ Hence, HMC3 was used as a model to test the effect of NPCBD in microglia, as was done in THP-1. Briefly, HMC3 cells were exposed to 100 ng/mL LPS or NPCBD suspension at concentrations ranging from 0.1 mg/mL to 0.005 mg/mL (Figure 3). Similarly to what was observed in THP-1, only the highest concentration of NPCBD showed a significant reduction of 28% (average of 2.21 μg/mL for the healthy group vs 1.59 μg/mL in the case of 0.1 mg/mL NPCBD treated group) in dsDNA concentration, while no differences were observed in terms of metabolic activity. Furthermore, of all the tested concentrations, NPCBD exhibited a similar cytokine release profile to untreated healthy cells, with a significantly higher release of every cytokine from the LPS-treated cells (Figure 3D–L). Microglia are the main immune cell of the CNS, and they become activated under pathological conditions.^26^ The effect of CBD on healthy rodent microglia (BV-2 cell line) has been recently reported showing no effect on Iba-1 expression when administered in doses of 1 μM, in correlation with enhanced levels of Mitofusin-2 (Mfn2).^26^ Mfn2 is a mitochondrial fusion protein involved in the inflammatory response mediated by microglia, whose levels were enhanced after CBD administration. Interestingly, its knockdown abolished CBD’s anti-inflammatory effect, proving its role in alleviating the inflammatory response both *in vitro* and in an *in vivo* model of experimental autoimmune encephalomyelitis (EAE).^26^ Hence, our NPCBD exhibited a similar safe behavior in concentrations from 0.1 mg/mL (2 μM CBD), showing a promising therapeutic potential.

In conclusion, NPCBD did not seem to induce a detrimental inflammatory environment in terms of the cytokine release profile compared with the one induced by LPS treatment in THP-1 and HMC3. However, these models present the inherent limitations of any *in vitro* assessment, such as the controlled and isolated environment and the presence of just one cell population. In addition, the lack of debris and dead cell removal is a limitation of every *in vitro* model that could conceal a better view of the inflammatory outcome after treatment. However, taking this in mind, in the dose–response assessed *in vitro*, NPCBD cytokine release profile was closer to the healthy control group, especially in HMC3, but also in the THP-1 model, exhibiting a promising behavior over the two main immune cell types they will interact with the real *in vivo* scenario.

### 3.3 NPCBD Ameliorated OGD-Induced Cytotoxicity

To overcome some of the limitations described above, NPCBD was tested in a complex model involving all of the relevant cell populations found in the *in vivo* scenario. Various *in vitro* models have been used over the last three decades to mimic stroke.^27^ The OGD model has proved to be the most reliable and reproducible.^28,29^ However, it is not possible to fully reproduce *in vitro* the high complexity found in the poststroke brain tissue. To overcome this limitation, diverse coculture models have been optimized to provide more complexity to the information provided by the *in vitro* model.^30^ Hence, a wide variety of cell populations, such as neurons, microglia, astrocytes, endothelial cells, or monocytes, among others, have been cocultured with this purpose.^30,31^ The primary cortical cells offer a more reliable model involving an intricately mixed population of cells, including microglia, astrocytes, neurons, and oligodendrocytes, among others. A PCC model of spinal cord injury (SCI) was recently described by our group as a platform to study acute and chronic inflammatory phases of SCI.^32^ While immortalized cell lines are more proliferative and easier to maintain, primary cells are considered more physiologically relevant.

**Figure 4:**
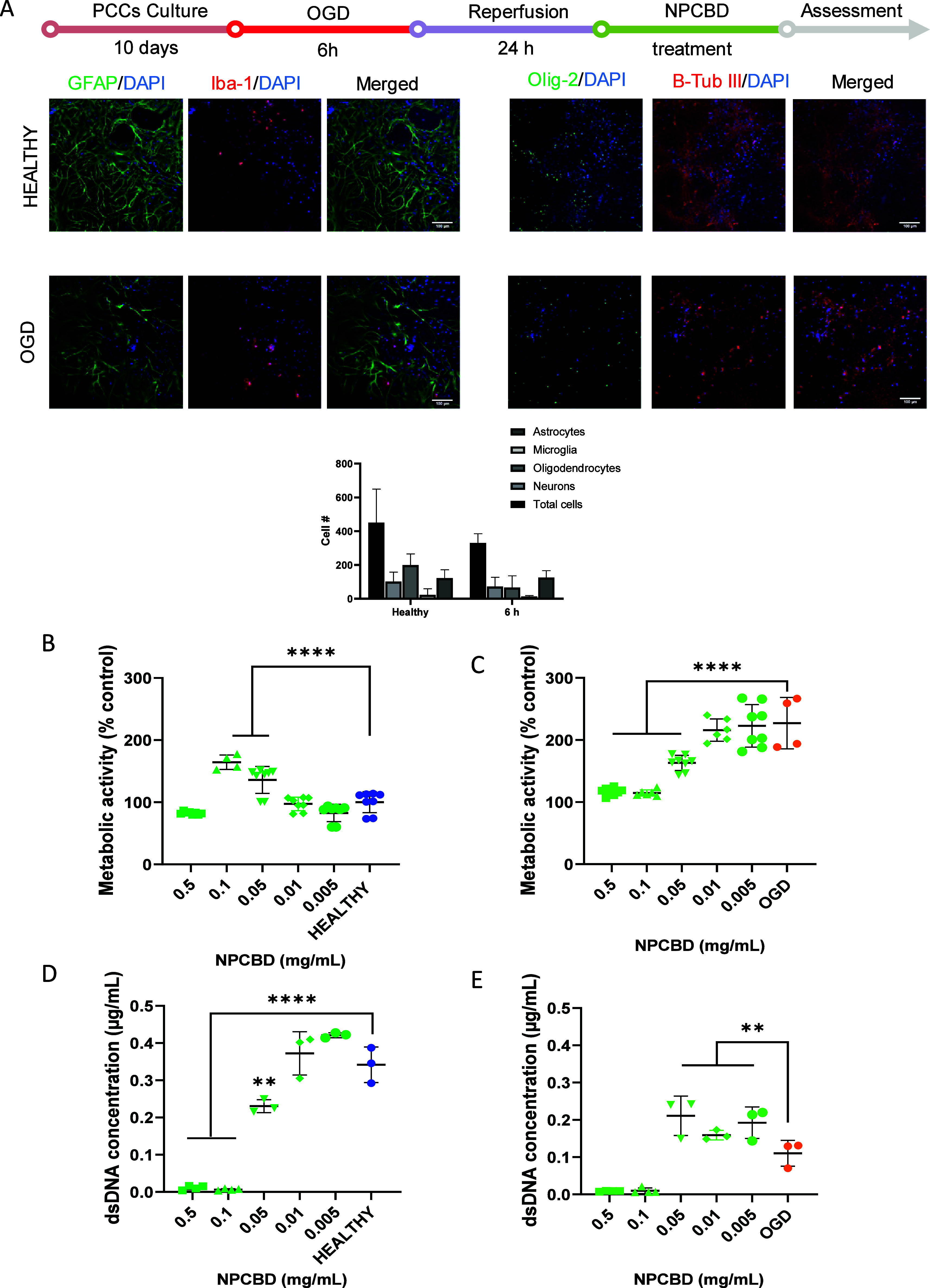
NPCBD restored dsDNA concentration after 6 h OGD and reperfusion model of rat primary cortical cells. (A) Experimental workflow of the PCC OGD *in vitro* model, immunocytochemistry after the reperfusion step, against the glial fibrillary acidic protein (GFAP) (astrocytes), Iba-1 (microglia), β-III-tubulin (β-Tub III) (neurons), and Olig-2 (oligodendrocytes) and its relative presence in the PCC quantified (data extracted from 5 pictures from 4 independent samples, *N* = 4). Data presented as the mean ± SD. Scale bar = 100 μm. dsDNA concentration was determined by Picogreen analysis, and metabolic activity of the culture was determined by alamarBlue after 3 days of treatment with NPCBD in healthy conditions (B and D) or after OGD exposure (C and E). Data are represented as mean ± SEM, *N* ≥ 4 experimental replicates. *p < 0.05, **p < 0.01, ***p < 0.001 vs Healthy or OGD cells. One-way ANOVA followed by Dunnet’s posthoc test.

Here, PCCs were optimized and implemented to test the NPCBD therapeutic effect in a relevant OGD model of ischemia (Figure 4A). Afterward, cells were exposed to OGD over 6 h, followed by 24 h of reperfusion as previously described.^28^ PCCs were analyzed in terms of dsDNA content and metabolic activity to ensure a balance of having enough damage to appreciate differences among treatments without compromising the viability of the whole culture. Metabolic activity and dsDNA content of PCCs were determined at 3 (Figure 4B–E) and 7 days (Figure S1) after NPCBD treatment. As expected, significant differences among groups were seen especially in metabolic activity that was considerably higher (>200%) in all the OGD-exposed groups and their dsDNA content significantly lower, with a decrease to an average of 33% viability (average of 0.34 μg/mL for healthy group vs 0.11 μg/mL in the case of OGD group). However, most NPCBD concentrations tested exhibited a similar value to the healthy cells. More importantly, the dsDNA concentration was significantly higher in NPCBD-treated groups after OGD, a 200% increase compared to that of the OGD groups (0.11 μg/mL in the OGD group vs 0.2 μg/mL for the NPCBD treated groups), suggesting a cytoprotective and regenerative effect 3 days after the treatment (Figure 4E). These results align with previous reports using CBD in similar concentrations (from 1 to 10 μM) *in vitro*(33) and *ex vivo*(34) OGD models. Moreover, some reports have shown cellular protection after OGD exposure by pretreating cells with CBD at even lower doses (from 0.1 to 1 μM).^35,36^ Interestingly, our results showed a great performance of the NPCBD in restoring PCC viability after an OGD insult, even at 0.01 mg/mL doses (corresponding to 0.2 μM), exhibiting great therapeutic potential.

Seven days after the NPCBD treatment, group differences were notably reduced (Figure S1). However, differences among the OGD-exposed and unexposed groups were still appreciable. Furthermore, the same statistically significant upper trend in dsDNA concentration was appreciated among OGD-exposed groups that received NPCBD afterward (Figure S1D). All in all, these results evidenced the potential of NPCBD to provide a friendly milieu for CNS cells and exhibit a potential cytoprotective effect after OGD.

### 3.4 NPCBD Treatment Ameliorates OGD/R-Induced Mitochondrial Impairment Both in HMC3 and PCC

The brain, known for its exceptionally high energy consumption, heavily relies on proper mitochondrial function. During a stroke, the ischemic event induces mitochondrial dysfunction, impeding the electron transport chain. Consequently, adenosine triphosphate (ATP) production is hindered, leading to an overexpression and accumulation of reactive oxygen species (ROS).^37^ Upon reperfusion in stroke, the restored blood and oxygen supply trigger oxidative damage and cell death through increased generation of mitochondrial ROS.^38^ Thus, it becomes imperative to investigate the impact of NPCBD on mitochondrial physiology and its potential to alleviate the effects of OGD-induced damage.

**Figure 5:**
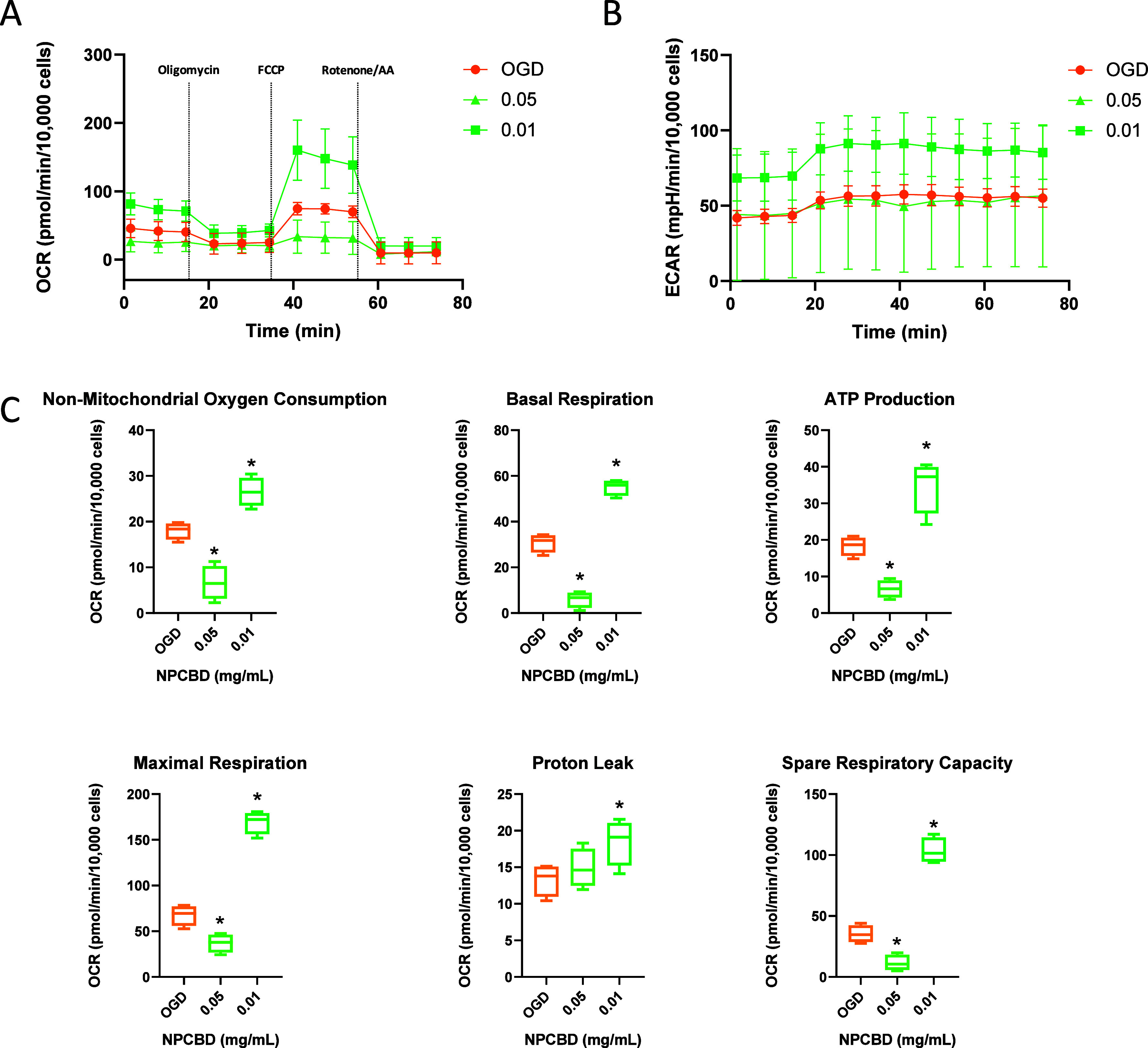
Mitochondrial dysfunction in HMC3 after OGD was ameliorated by NPCBD treatment. Cell Mito Stress analysis of mitochondrial respiratory capacity in HMC3 cells in the OGD model. Cells were incubated 30 min before the experiment in an XF assay medium supplemented with 5 mM glucose and 2 mM glutamine and subsequently injected with oligomycin (1 μM), FCCP (0.5 μM), antimycin (1 μM), and rotenone (1 μM). Continuous OCR values (pmol/min/10,000 cells) (A) ECAR values (mpH/min/10,000 cells) (C) and OCR parameters 3 days after the treatment are reported (*N* ≥ 3, *p < 0.05). One-way ANOVA followed by Dunnet’s posthoc test

**Figure 6:**
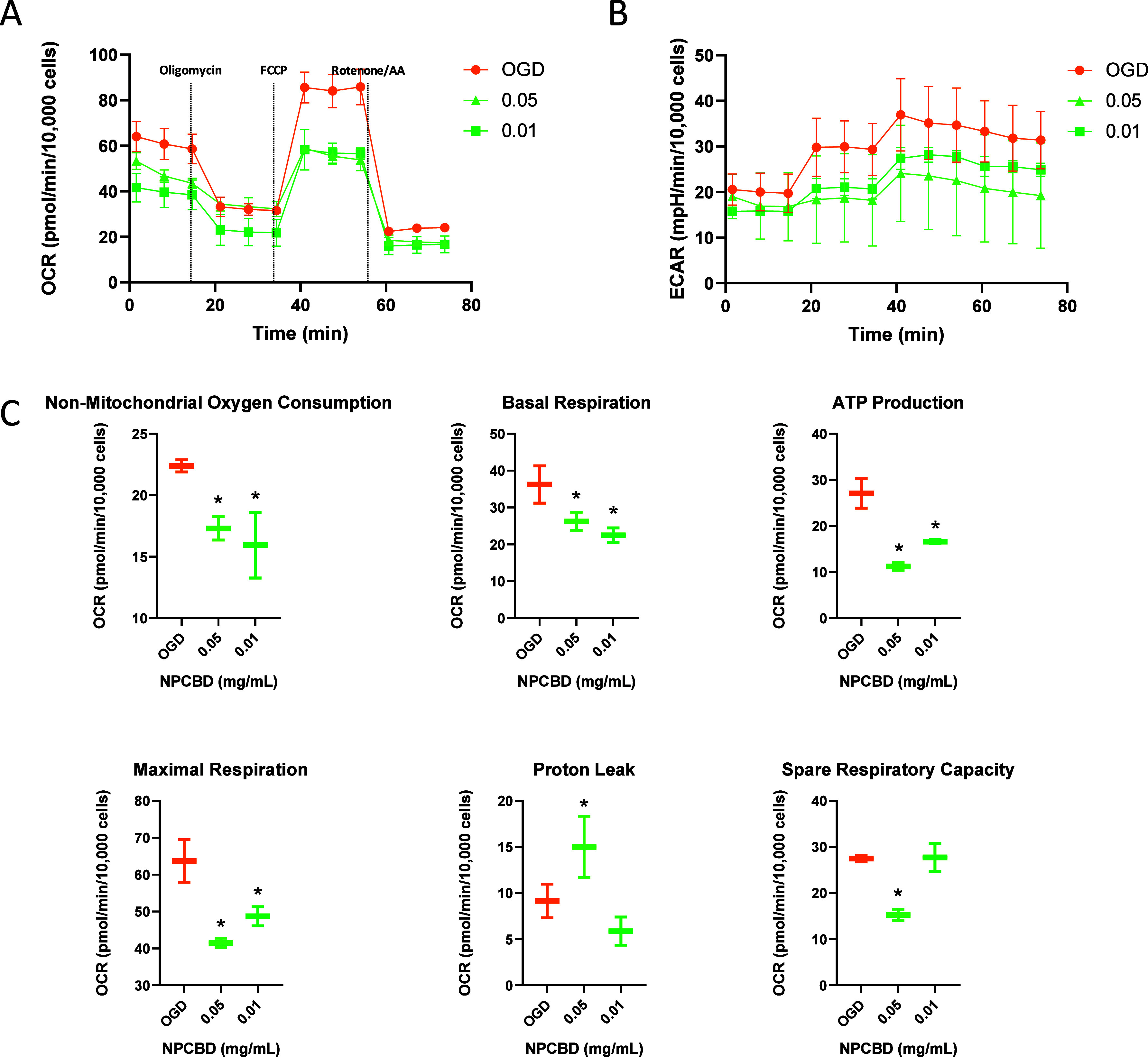
Mitochondrial dysfunction in PCC after OGD was ameliorated by NPCBD treatment. Cell Mito Stress analysis of mitochondrial respiratory capacity in PCC cells in the OGD model. Cells were incubated 30 min before the experiment in an XF assay medium supplemented with 5 mM glucose and 2 mM glutamine and subsequently injected with oligomycin (1 μM), FCCP (1.5 μM), antimycin (1 μM), and rotenone (1 μM). Continuous OCR values (pmol/min/10,000 cells) (A), ECAR values (mpH/min) (B), and OCR parameters (C) 3 days after the treatment are reported (*N* ≥ 3, *p < 0.05). One-way ANOVA followed by Dunnet’s posthoc test

Mitochondrial performance was assessed by measuring the oxygen consumption rate (OCR) and extracellular acidification rate (ECAR) using the Mito Stress test under both healthy and OGD conditions in the HMC3 cell line (Figure 5) and PCC (Figure 6). The titration of FCCP, an uncoupling agent used to determine maximal respiration and calculate spare respiratory capacity, was initially assessed to optimize the dosage for each cell culture (Figures S2 and S3). OCR values directly relate to the mitochondrial electron transport rate, while ECAR correlates with metabolic activity.^32^ Considering previous results suggesting that lower concentrations of NPCBD exhibit more favorable performance over PCCs, all subsequent tests were conducted using only 0.05 and 0.01 mg/mL, corresponding to 1.00 μM and 0.20 μM CBD, respectively. The lowest concentration of 0.005 mg/mL (equivalent to 0.10 μM CBD) showed no differences from the 0.01 mg/mL concentration and was thus excluded from further assessments.

During an OGD insult, astrocytes and microglia become reactive due to massive cell death, leading to impaired mitochondrial function.^33^ To discern the effects of NPCBD on mitochondrial activity, we evaluated mitochondrial respiration in healthy cells, finding no statistically significant differences compared with the healthy group (Figure S4) across all measured parameters. However, the OGD/R insult on HMC3 cells caused a significant decline in basal respiration compared with the healthy control (Figure 5). Notably, at the lower NPCBD dose (0.01 mg/mL, corresponding to 0.20 μM of CBD), OCR values affected by the OGD insult were restored, closely resembling those of the untreated (HEALTHY) cells. These findings align with previous studies using CBD at similar doses in cell lines such as hippocampal neurons (HT22 cells)^33^ and BE(2)-M17 neuroblastoma cells.^39^

Furthermore, our data suggest a dose-dependent effect of NPCBD on HMC3, with the higher dose not ameliorating OGD-induced mitochondrial dysfunction. This aligns with previous findings demonstrating the dose-dependent nature of CBD’s effects.^39^ ECAR, an indicator of glycolysis, was also impaired after the OGD insult, showing some recovery after NPCBD treatment at 0.01 mg/mL, although the differences were not statistically significant (Figure 5B).

The ability of NPCBD to counteract mitochondrial damage induced by the OGD insult was also assessed in PCC (Figure 6). Interestingly, an increase in both OCR and ECAR values was observed after the OGD insult, potentially resulting from microglia and astrocyte activation.^32^ At the higher dose of 0.05 mg/mL, the values in healthy cells were similar to those exhibited by the group treated with OGD, suggesting a potential effect on the activation of neuroglia cells (Figure S5), while the lower dose exhibited a profile similar to untreated cells. NPCBD significantly ameliorates mitochondrial dysfunction at both doses, as indicated by the reported OCR values in Figure 6C. Thus, a significant decrease in all tested parameters can be observed in groups receiving NPCBD after the OGD insult, suggesting the treatment’s ability to restore mitochondrial activity to levels close to those of the control group.

The effects of OGD on HMC3 monoculture did not mirror the data obtained from PCC coculture. A potential explanation is the cell communication within the PCC, which is more representative of cortical response in vivo, incorporating compensatory mechanisms in the mixed population. These considerations are crucial given CBD’s promiscuity in its action on different molecular targets. Similar results have been reported in other studies employing monocultures and cocultures.^40^ Overall, results from Seahorse analysis indicate a neuroprotective effect provided by NPCBD, reducing OGD-induced oxidative stress, in line with previous findings.^33,39^

### 3.5 NPCBD Successfully Ameliorated Inflammation Related to Hypoxic Conditions After OGD Exposure

**Figure 7:**
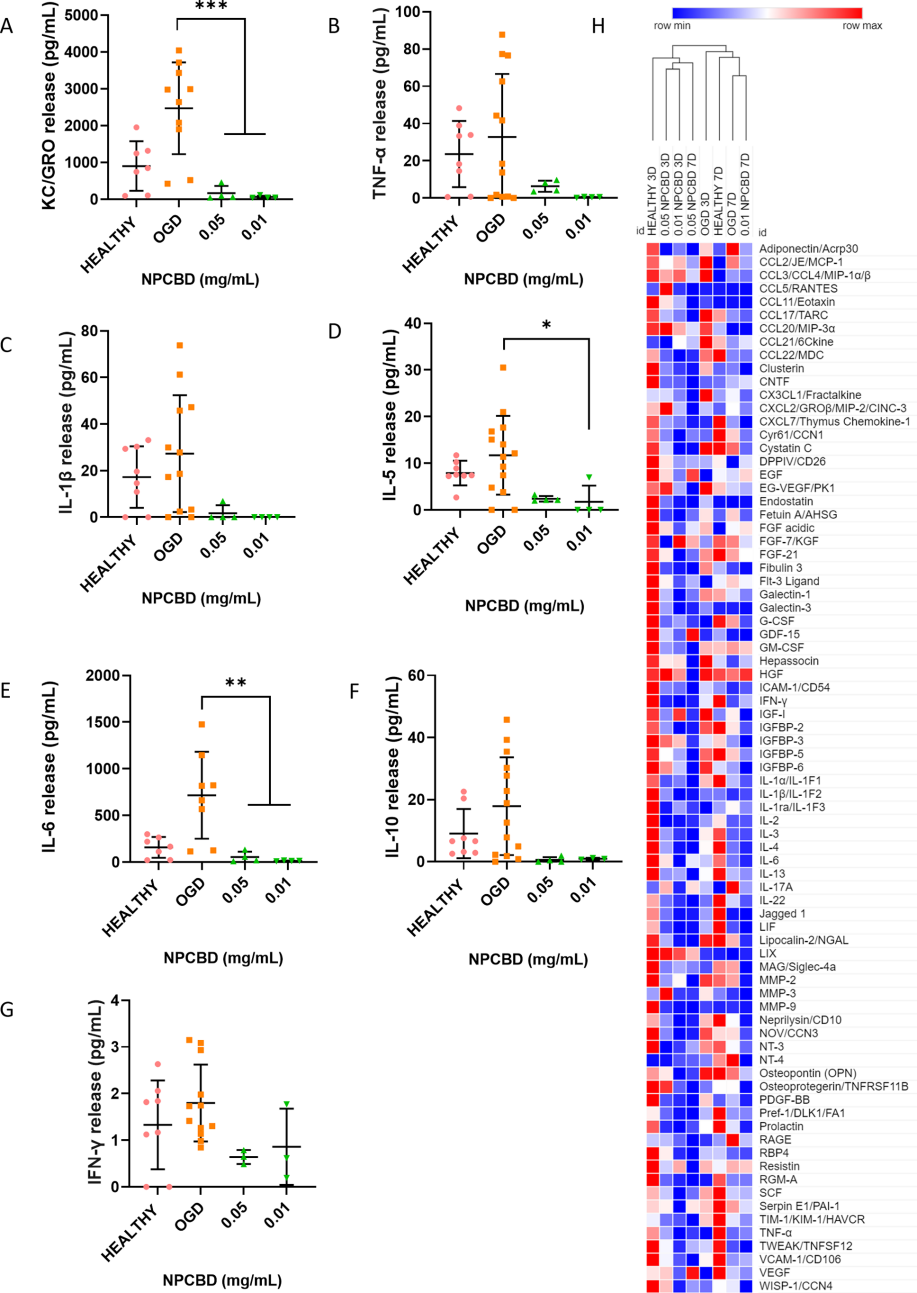
Inflammatory response of PCC after OGD and NPCBD treatment. Determination of the release of pro-inflammatory (A–E), anti-inflammatory (F), and dual (G) cytokines from the PCC after 24 h of treatment with NPCBD at two different doses. Cytokine concentration released to the supernatant was analyzed by multiplex ELISA. Data are represented as mean ± SEM, *N* ≥ 4 experimental replicates. *p < 0.05, **p < 0.01, ***p < 0.001 vs Healthy cells. One-way ANOVA followed by Tukey’s posthoc test. (H) Hierarchical clustering analysis of 79 analytes. The mean pixel density was analyzed by the proteome profile array of proteins secreted by PCC in the supernatant. Colors define activation as highly expressed (red) and no expression (blue). Treatment was given at two different doses after OGD exposure, and the supernatant was analyzed at two time points, day 3 and day 7. The experiment was carried out in three biological replicates, and supernatants were pooled together to perform the proteome profiler array. Each analyte on the array was printed in duplicate. The values shown per time point are an average of both.

Stroke presents a highly complex pathophysiology.^27^ The innate immune system is activated after ischemic insult due to the accumulation of cell debris and dying neurons. Hence, the primary response is led by microglia, the resident immune population of the CNS, which are the first population to become activated, and monocytes from the peripheral immune system, differentiated into macrophages (that migrate into the ischemic site).^24^ After their activation, both cell populations can polarize into phenotypes capable of promoting or ameliorating the inflammatory response.^24^ After the ischemic event, microglia and astrocytes become reactive, producing cytokines, chemokines, and growth factors.^41^ Hence, both neuroglial cells lead to secondary neuroinflammation that promotes further injury, which results in cell death and promotes recovery. These proinflammatory signals from the resident immune cells induce the infiltration of a wide range of inflammatory cells such as monocytes, neutrophils, or T cells. Hence, the secretion of some of the critical cytokines associated with ischemic stroke was assessed 24 h after NPCBD treatment (Figure 7A–G). Furthermore, a deeper analysis of the inflammatory state of the PCCs was assessed 3 and 7 days after OGD exposure and NPCBD treatment through proteome profiler analysis (Figure 7H). After 24 h of NPCBD treatment, the level of secreted pro-inflammatory cytokines was higher in the OGD than in the others, being statistically significant only in the case of KC/GRO, 90% decrease compared to the OGD group (2400 pg/mL in the OGD group versus 200 pg/mL in NPCBD treated groups), a decrease of 75% of IL-5 (11.4 pg/mL on average for the OGD group versus 3 pg/mL in NPCBD treated groups), and a decrease of 93% of IL-6 (714 pg/mL in the OGD group versus 55 pg/mL in NPCBD treated groups). It can be noticed that KC/GRO release (also known as CXCL1) was ten times higher in OGD-exposed cells than in the groups treated afterward with NPCBD (Figure 6A). This was also observed in the heatmap representing the relative expression of cytokines, when an increase in the relative expression of CXCL1 was seen for the OGD-exposed groups at 3 days, slightly decreasing at 7 days. It has been previously reported that neutrophil infiltration occurs as a response to increased levels of chemokines in the damaged tissue, including CXCL-1 chemokine. Moreover, inhibiting CXCL-1/CXCR2 (CXC group receptor 2) activation dramatically reduced the inflammatory response after stroke and the lesion area.^42^

On the contrary, it was reported that IL-5, upregulated here in the OGD-exposed group, potentially has a protective role on patients of acute ischemic stroke.^43^ Thus, the upregulation seen here (Figure 7D) is related to the internal mechanism of protection mediated by microglia and astrocytes to induce a protective effect during the acute phase after ischemic insult. Furthermore, IL-6 is one of the critical factors that is upregulated after ischemic stroke.^44^ After OGD exposure, a significant increase in IL-6 secretion was detected with values seven times higher than the healthy group, and a subsequent reduction after NPCBD treatment was noticed (Figure 7E). IL-6 leads to the activation and recruitment of neutrophils and monocytes and enhances local inflammatory response.^45^ The secretion of other crucial markers of ischemic stroke was relatively reduced after NPCBD treatment, as seen in the heatmap. The tumor necrosis factor receptor superfamily member 11B (TNFSF11), which is essential for immune system regulation, showed similar values to the healthy control after the treatment with 0.05 mg/mL NPCBD (Figure 7H). CCL21, a small cytokine that belongs to the CC chemokine family, was upregulated 3 days after OGD, while the values after NPCBD treatment were similar to healthy cells. CCL21 has been reported to be upregulated after ischemic events, such as limb ischemia, and regulates migration and homing of T lymphocytes via CCR7.^46^ All of these cytokines are consistently secreted by reactive microglia and astrocytes in response to ischemic events and are often used as biomarkers to indicate neuroinflammatory or neurodegenerative disorders. A hierarchical cluster analysis was performed on the results obtained for all of the treated groups, showing two clusters in terms of similarity. One includes Healthy 3D, NPCBD 3D (both concentrations tested), and 0.05 mg/mL NPCBD 7D. Hence, a similar inflammatory profile to untreated healthy cells, in terms of secreted cytokines, was noticed by NPCBD-treated cells after OGD exposure, exhibiting a promising potential to address the inflammatory response in the subacute phase after the ischemic insult.

The effect of NPCBD on OGD-related inflammation is consistent with many previous studies using CBD in classical formulations.^13,26,35,36^ Neuroinflammation is pivotal in secondary injury after cerebral ischemia-induced brain injury. Thus, a reduction in the main pro-inflammatory cytokines and chemokines after the treatment with NPCBD proves its potential as a new CBD formulation to ameliorate ischemic-related inflammatory processes in the CNS. The mechanism of action of CBD is still a matter of discussion nowadays. The endocannabinoid receptor CB_2_ is principally expressed in immune cells, including microglial cells. It can be found in other cell types, such as fibroblasts and chondrocytes, being a peripheral cannabinoid receptor.

Moreover, it was described as present in nervous tissues, such as dorsal root ganglia or cochlear inner hair cells.^47^ We hypothesized that the effect of the NPCBD would be through CB_2_ interaction, and to prove it, we blocked the receptor with the selective CB_2_ inverse agonist SR144528. However, no significant differences were appreciated in terms of metabolic activity, dsDNA content, or cytokine release (data not shown). Hence, we could not confirm whether the NPCBD system acted through this receptor. Since CBD is a promiscuous drug capable of interacting with different receptors in the endocannabinoid system, some of which belong to the family of G-protein-coupled receptors, there are many possible routes for NPCBD to act. Previous reports showed a partial reduction of CBD protection after OGD on pericytes by 5HT_1A_ antagonism.^48^ According to the authors, CBDs can activate 5HT_1A_, the molecular target underpinning CBD’s therapeutic effect on stroke. The role of 5-HT_1A_ and PPARγ was also reported in the context of the permeability of the BBB, which was increased by CBD treatment.^40^

Moreover, it has been reported that CB_2_ and 5HT1A receptors may interact to form macromolecular complexes.^49^ In a rodent model, a significant reduction in the expression of CB_2_–5HT_1A_-Hets in CBD-treated rats has been reported.^49^ Altogether, this supports the hypothesis of the pivotal role of 5HT_1A_ in the CBD-mediated amelioration of the OGD damage. The pathology of ischemic stroke is multifaceted, which makes a promiscuous drug, CBD, a potential candidate for success compared with compounds aimed at a single target.^40^

### 4 Conclusions

The primary objective of this investigation is to confirm the safety and efficacy of NPCBD *in vitro* by using two relevant human immune cell lines. Furthermore, we aim to explore the potential of CBD in treating ischemic events and compare its effects to those of traditional formulations. In this study, we present the performance of a well-known polymeric nanocarrier, PLGA, in delivering the highly hydrophobic drug CBD to address neuroinflammation associated with ischemic stroke. Given the favorable regulatory history and excellent properties of PLGA for hydrophobic drug delivery, our NPCBD demonstrates enhanced potential for successful translation to preclinical and clinical settings.

The physicochemical properties of our NPCBD formulation, including hydrodynamic diameter, zeta potential, polydispersity, and stability under physiological conditions, make it a potential candidate for intravenously targeting inflammation. CBD’s high loading efficiency within the PLGA matrix is also a significant advantage. In addition, the *in vitro* OGD model used to test the formulation’s ability to alleviate the inflammatory response was promising, demonstrating a restorative effect on primary cortical cultures. The Mito Stress studies also showed the formulation’s ability to restore mitochondrial function in both HMC3 and PCC cultures at doses of 1 and 0.2 μM of CBD, which is a critical factor in ameliorating OGD-induced mitochondrial dysfunction. However, further studies are needed to assess the therapeutic effects of NPCBD in an appropriate *in vivo* model of an ischemic stroke.

The use of an *in vitro* model based on primary cortical cultures for the purpose of robust stroke emulation presents both advantages and challenges. On the one hand, the complexity of multiple coexisting cell populations contributes to the robustness of the model. However, this same feature also increases the variability, making it difficult to distinguish statistically significant differences. In this study, we confirmed that the CBD polymeric nanoformulation exhibits physicochemical properties that can address the inherent solubility problems of CBD while maintaining its bioactivity and expanding its potential for intravenous therapeutic use. Furthermore, the ability of NPCBD to reduce the inflammatory response induced by ischemic conditions such as ischemic OGD was demonstrated in an *in vitro* model that incorporates mixed populations from the central nervous system. In conclusion, we have developed, characterized, and evaluated a straightforward CBD nanoparticulate polymeric delivery system *in vitro* to address neuroinflammation caused by ischemic stroke.

## Supporting Information Available

Variability of NPCBD physicochemical properties among batches (Table S1); effect of 7 day NPCBD treatment after a 6 h OGD and reperfusion model of rat primary cortical cells (Figure S1); FCCP optimization with the XF Cell Mito Stress Test on HMC3 (Figure S2); FCCP optimization with the XF Cell Mito Stress test on PCC (Figure S3); Cell Mito Stress analysis of mitochondrial respiratory capacity in HMC3 cells under normoxia (Figure S4); Cell Mito Stress analysis of mitochondrial respiratory capacity in PCC cells under normoxia (Figure S5); the cytokine array detects changes in cytokines and chemokines in the media describing the inflammatory phenotype of PCC after the treatments (Figure S6); analysis of 79 analytes related to inflammation (Figure S7) (PDF)
