# Supplementary material for: Therapeutic Polymer-Based Cannabidiol Formulation: Tackling Neuroinflammation Associated with Ischemic Events in the Brain
Source: Mol Pharm. 2024 Feb 27;21(4):1609–24. doi: 10.1021/acs.molpharmaceut.3c00244 (PMC10988560; doi:10.1021/acs.molpharmaceut.3c00244)
Supplement: Supplementary file 1 — mp3c00244_si_001.pdf [file mp3c00244_si_001.pdf]

SUPPORTING INFORMATION

Therapeutic polymer-based Cannabidiol formulation: Tackling neuro-inflammation associated with ischemic events in the brain

Merari Tumin Chevalier[1], Mansoor Al-Waeel [1], Amir M. Alsharabasy, Ana Lúcia Rebelo, Sergio Martin-Saldaña* and Abhay Pandit*

CÚRAM, SFI Research Centre for Medical Devices, University of Galway, Galway, H92 W2TY, Ireland

[1] These authors contributed equally to this work

*Corresponding author addresses: abhay.pandit@universityofgalway.ie (Prof Abhay Pandit); smartinsaldana@gmail.com (Dr Sergio Martin-Saldaña)

Table S1: Variability of NPCBD physicochemical properties among batches. Hydrodynamic diameter (Dh, by intensity), polydispersity index (PDI); and zeta potential values (δ).

BATCH #	CBD % w/w	Dh (nm)	PDI	δ (mV)
1	10	240.5 ± 5.9	0.22 ± 0.06	-21.0 ± 0.62
2		269 ± 5.9	0.16 ± 0.02	-26.3 ± 0.35
3		254.3 ± 0.6	0.13 ± 0.03	-31.0 ± 0.86
4		214.7 ± 2.1	0.07 ± 0.02	-18.1 ± 0.20

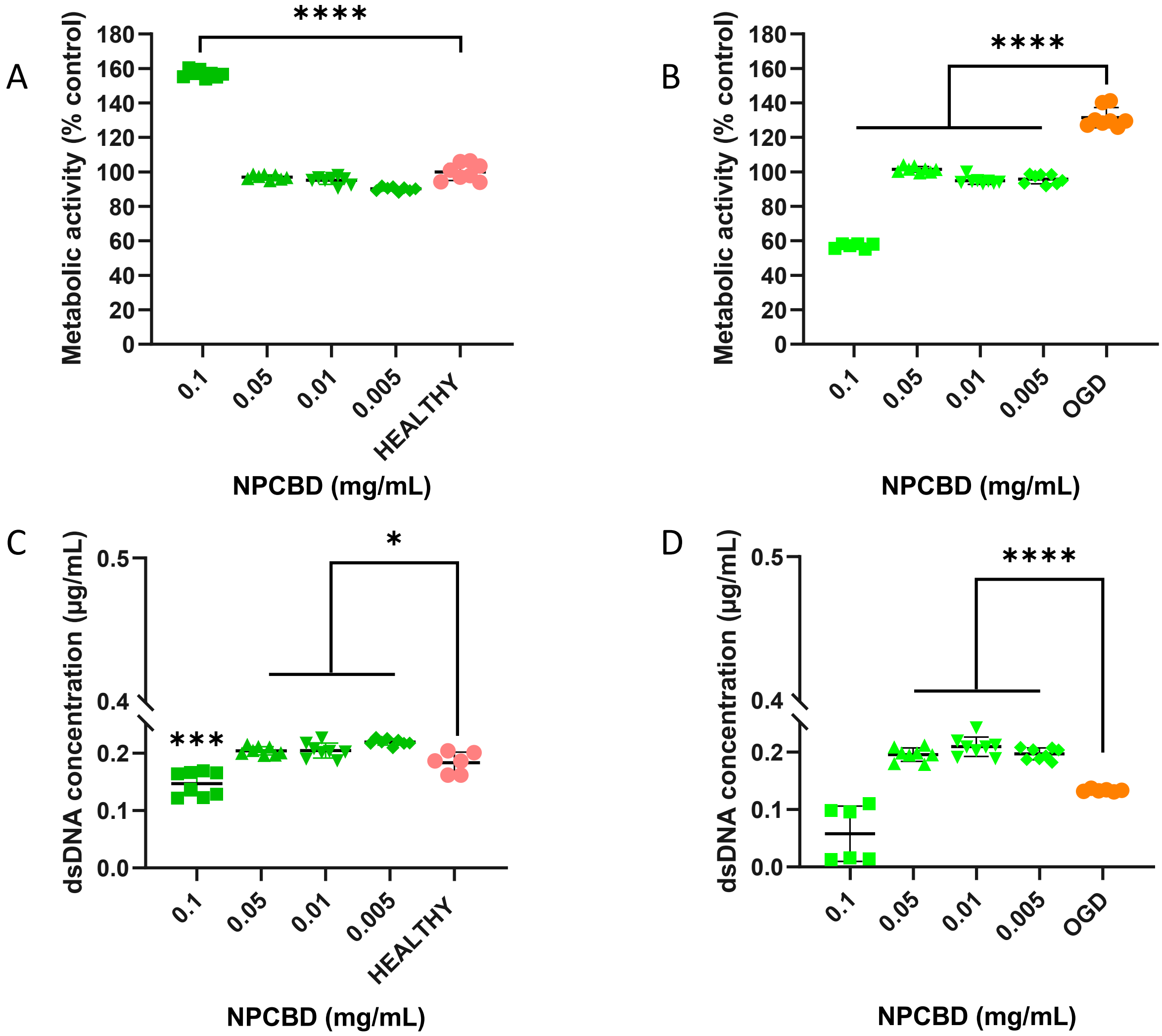

Figure S1. Seven days treatment NPCBD dose response after a six h OGD and reperfusion model of rat primary cortical cells. dsDNA concentration was determined by Picogreen™ analysis and metabolic activity of the culture was determined by alamarBlue™ after three days of treatment with NPCBD in healthy conditions (A and C) or after OGD exposure (B and D). Data are represented as mean ± SEM, N≥4 experimental replicates. *p<0.05, **p<0.01, ***p<0.001 vs Healthy or OGD cells. Ordinary One way ANOVA followed by Dunnet's post-hoc test.

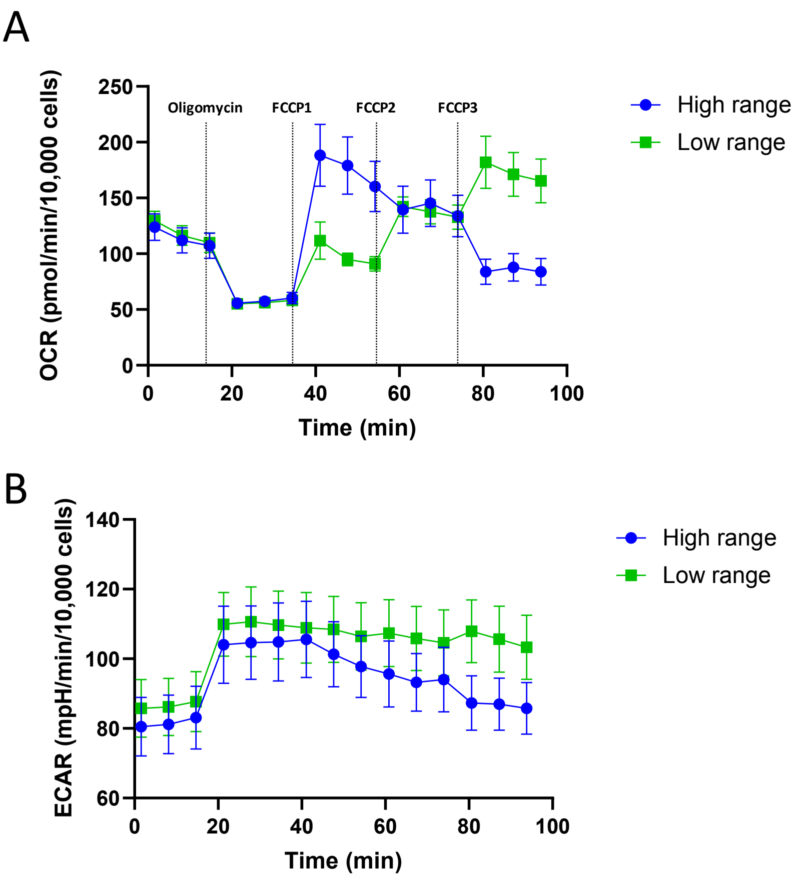

Figure S2. FCCP Optimization with the XF Cell Mito Stress Test on HMC3. Continuous (A) OCR values (pmol/min/10,000 cells) (B) ECAR values (mpH/min/10,000 cells). Data are represented as mean ± SEM, N≥3 experimental replicates.

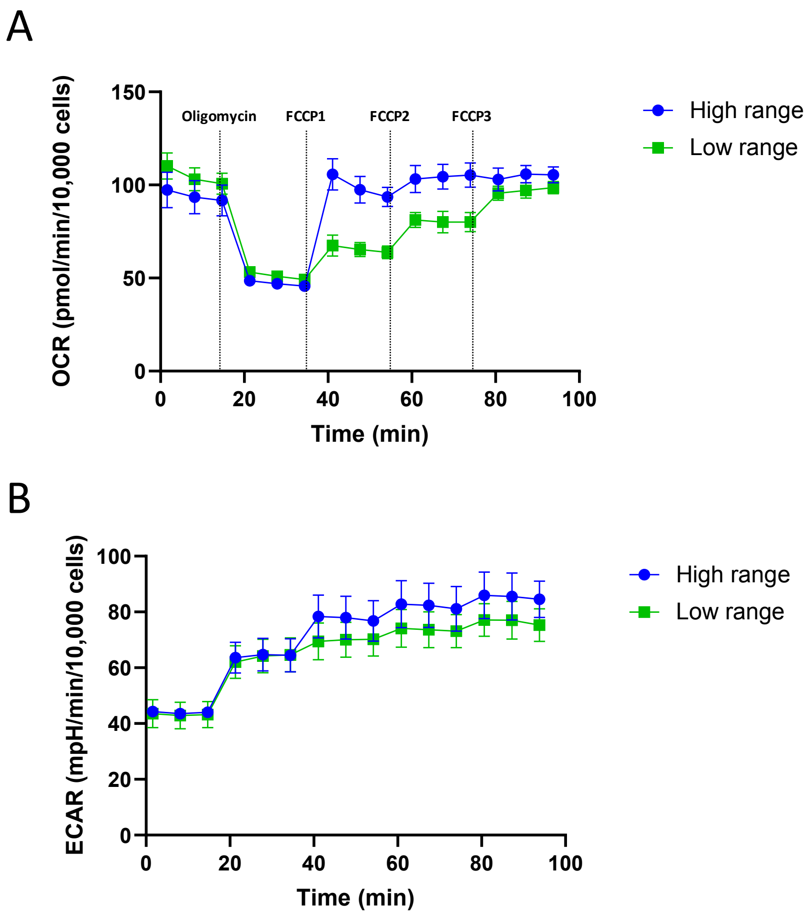

Figure S3. FCCP Optimization with the XF Cell Mito Stress Test on PCC. Continuous (A) OCR values (pmol/min/10,000 cells) (B) ECAR values (mpH/min/10,000 cells). Data are represented as mean ± SEM, N≥3 experimental replicates.

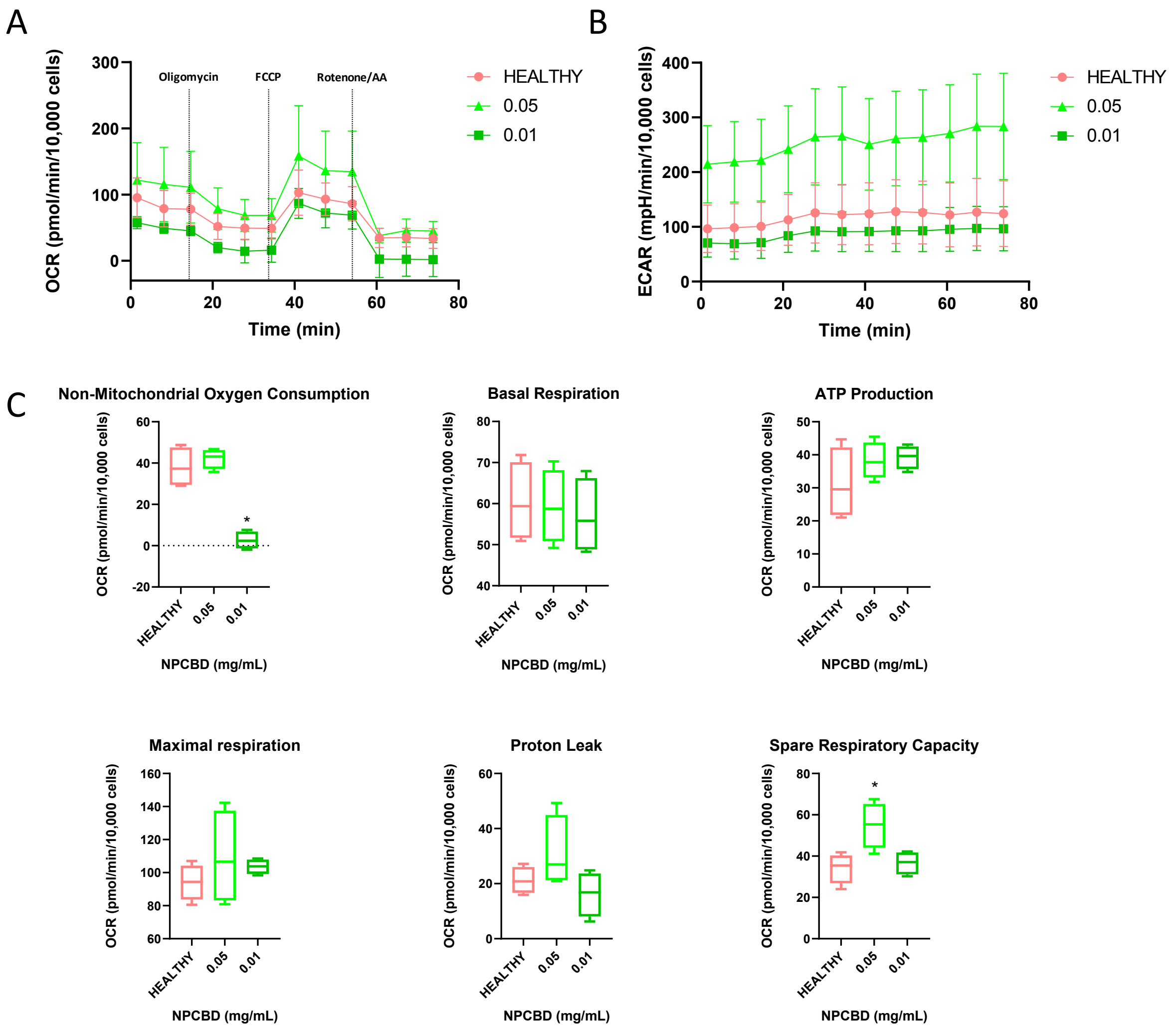

Figure S4. Cell Mito Stress analysis of mitochondrial respiratory capacity in HMC3 cells under normoxia. Cells were incubated 30 min before the experiment in an XF assay medium supplemented with 5 mM glucose and 2 mM glutamine and subsequently injected with oligomycin (1 μM), FCCP (1.5 μM), antimycin (1 μM) and rotenone (1 μM). Continuous (A) (A) OCR values (pmol/min/10,000 cells) (B) ECAR values (mpH/min/10,000 cells), (C) and OCR parameters three days after the treatment are reported. ($n \geq 3$, *$p < 0.05$).

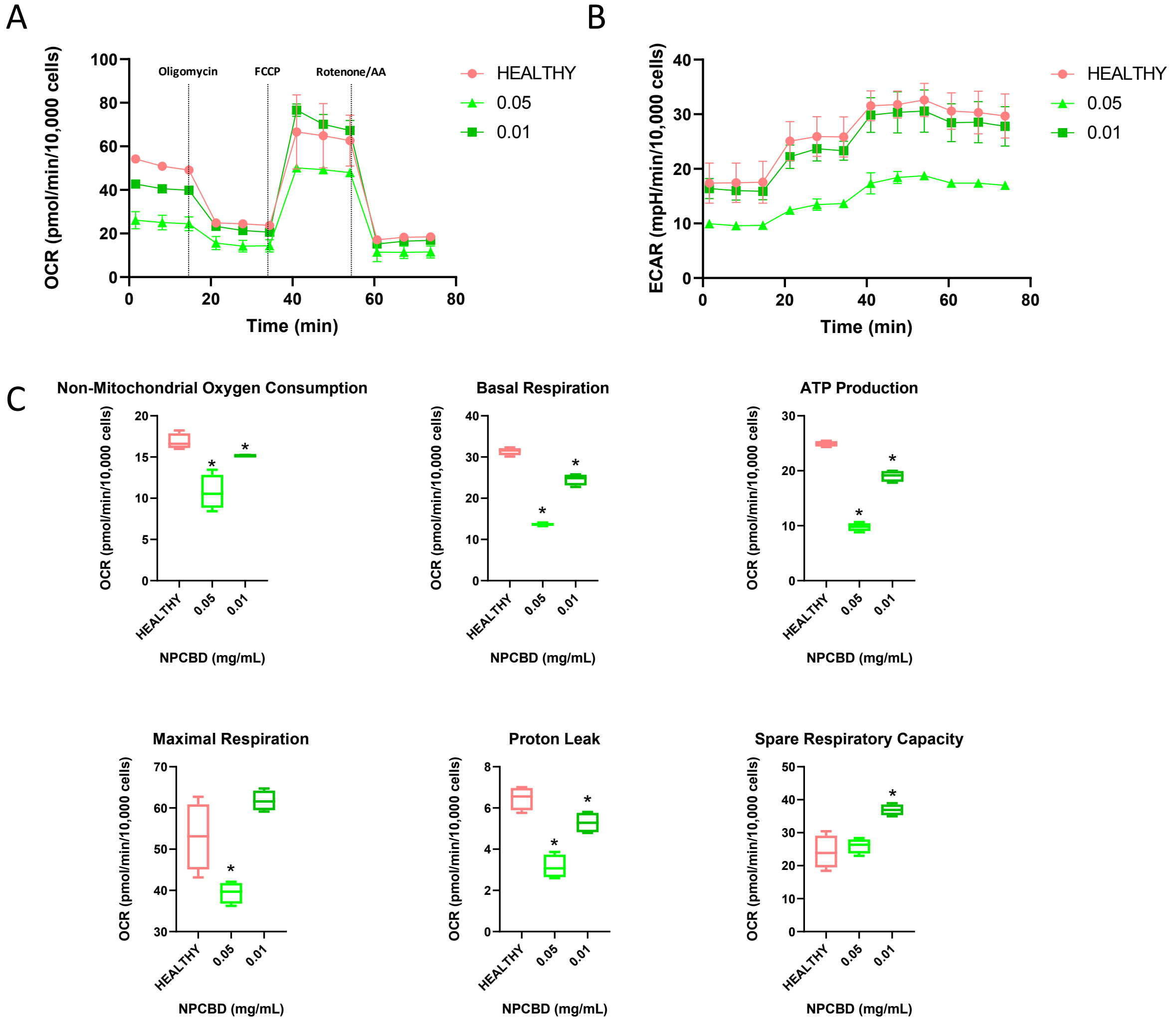

Figure S5. Cell Mito Stress analysis of mitochondrial respiratory capacity in PCC cells under normoxia. Cells were incubated 30 min before the experiment in an XF assay medium supplemented with 5 mM glucose and 2 mM glutamine and subsequently injected with oligomycin (1 µM), FCCP (1.5 µM), antimycin (1 µM) and rotenone (1 µM). Continuous (A) OCR values (pmol/min/10,000 cells) (B) ECAR values (mpH/min/10,000 cells), (C) and OCR parameters three days after the treatment are reported. ($n \geq 3$, *$p < 0.05$).

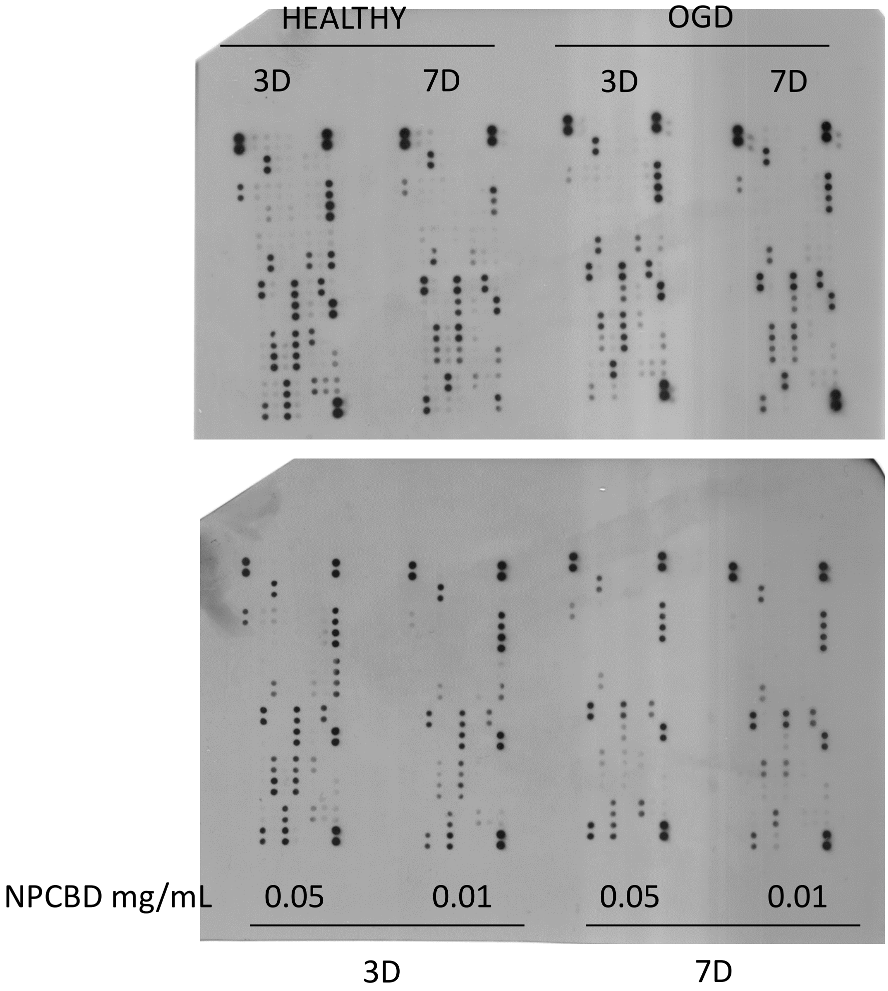

Figure S6. The Cytokine Array detects changes in cytokines and chemokines in the media describing the inflammatory phenotype of PCC after the treatments. Proteome profiler membranes of the assessed groups (Healthy, OGD, and OGD+NPCBD at 0.05 and 0.01 mg/mL) and at two timepoints (day-3, day-7). At least three experimental replicates were pulled supernatant and run for two technical replicates.

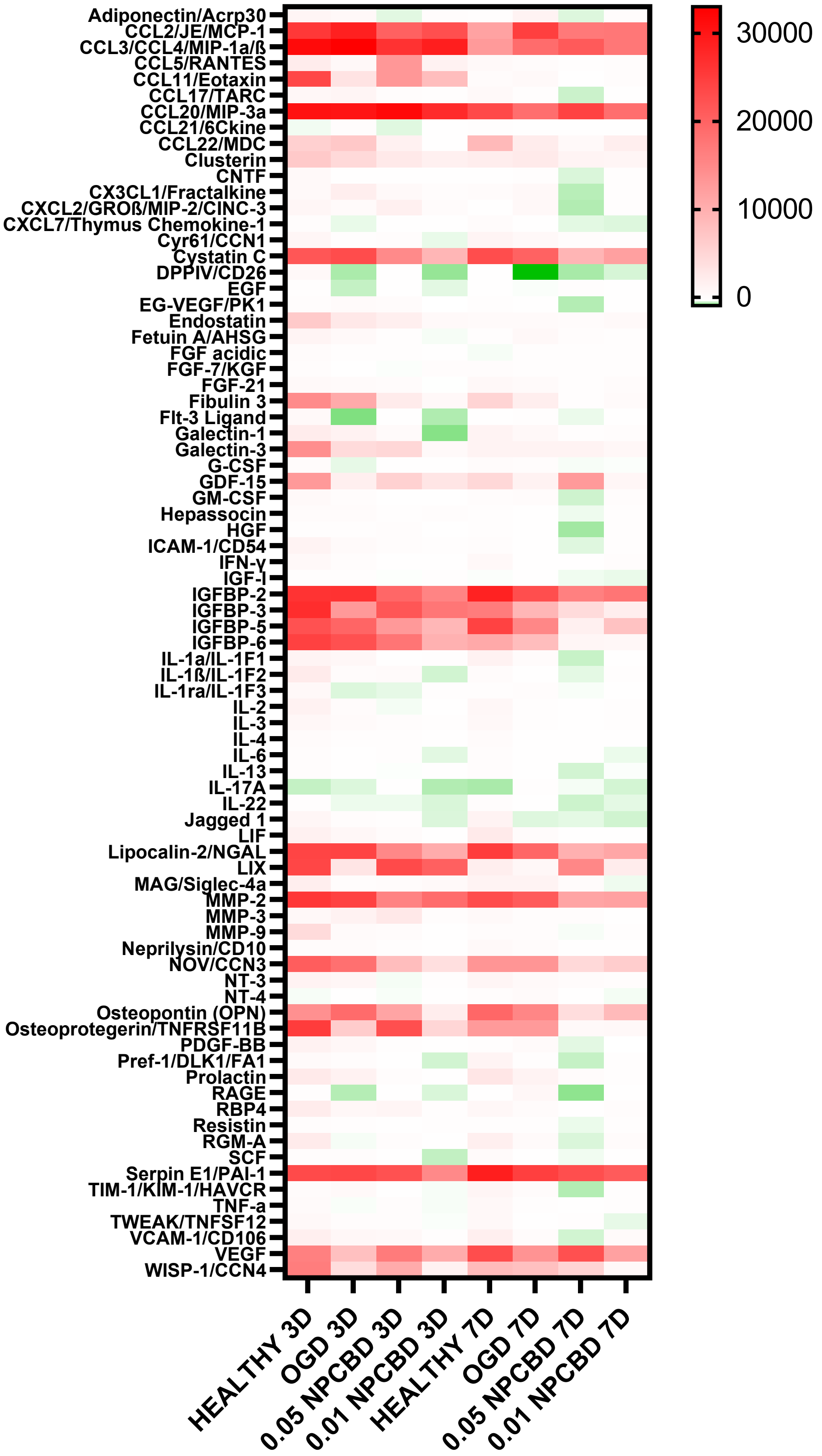
Adiponectin/Acrp30
CCL2/JE/MCP-1
CCL3/CCL4/MIP-1a/ß
CCL5/RANTES
CCL11/Eotaxin
CCL17/TARC
CCL20/MIP-3a
CCL21/6Ckine
CCL22/MDC
Clusterin
CNTF
CX3CL1/Fractalkine
CXCL2/GROß/MIP-2/CINC-3
CXCL7/Thymus Chemokine-1
Cyr61/CCN1
Cystatin C
DPPIV/CD26
EGF
EG-VEGF/PK1
Endostatin
Fetuin A/AHSG
FGF acidic
FGF-7/KGF
FGF-21
Fibulin 3
Flt-3 Ligand
Galectin-1
Galectin-3
G-CSF
GDF-15
GM-CSF
Hepassocin
HGF
ICAM-1/CD54
IFN-γ
IGF-I
IGFBP-2
IGFBP-3
IGFBP-5
IGFBP-6
IL-1a/IL-1F1
IL-1ß/IL-1F2
IL-1ra/IL-1F3
IL-2
IL-3
IL-4
IL-6
IL-13
IL-17A
IL-22
Jagged 1
LIF
Lipocalin-2/NGAL
LIX
MAG/Siglec-4a
MMP-2
MMP-3
MMP-9
Neprilysin/CD10
NOV/CCN3
NT-3
NT-4
Osteopontin (OPN)
Osteoprotegerin/TNFRSF11B
PDGF-BB
Pref-1/DLK1/FA1
Prolactin
RAGE
RBP4
Resistin
RGM-A
SCF
Serpin E1/PAI-1
TIM-1/KIM-1/HAVCR
TNF-a
TWEAK/TNFSF12
VCAM-1/CD106
VEGF
WISP-1/CCN4
HEALTHY 3D
OGD 3D
0.05 NPCBD 3D
0.01 NPCBD 3D
HEALTHY 7D
OGD 7D
0.05 NPCBD 7D
0.01 NPCBD 7D
30000
20000
10000
0

1

2

Figure S7. Analysis of 79 analytes related to inflammation. The mean pixel density was analyzed by the proteome profile array of proteins secreted by PCC in the supernatant. Colors define the semi-quantitative values related to protein activation as highly expressed (red) and no expression (green). Treatment was given at two doses after OGD exposure, and the supernatant was analyzed at two time points, day three and day seven. The experiment was carried out in three biological replicates, and supernatants were pooled together to perform the proteome profiler array. Each analyte on the array was printed in duplicate. The values shown per time point are an average of both.
